# A Probabilistic Boolean Network Approach for the Analysis of Cancer-Specific Signalling: A Case Study of Deregulated PDGF Signalling in GIST

**DOI:** 10.1371/journal.pone.0156223

**Published:** 2016-05-27

**Authors:** Panuwat Trairatphisan, Monique Wiesinger, Christelle Bahlawane, Serge Haan, Thomas Sauter

**Affiliations:** 1 Systems Biology group, Life Sciences Research Unit, University of Luxembourg, Luxembourg, Luxembourg; 2 Molecular disease mechanisms group, Life Sciences Research Unit, University of Luxembourg, Luxembourg, Luxembourg; University of Erlangen-Nuremberg, GERMANY

## Abstract

**Background:**

Signal transduction networks are increasingly studied with mathematical modelling approaches while each of them is suited for a particular problem. For the contextualisation and analysis of signalling networks with steady-state protein data, we identified probabilistic Boolean network (PBN) as a promising framework which could capture quantitative changes of molecular changes at steady-state with a minimal parameterisation.

**Results and Conclusion:**

In our case study, we successfully applied the PBN approach to model and analyse the deregulated Platelet-Derived Growth Factor (PDGF) signalling pathway in Gastrointestinal Stromal Tumour (GIST). We experimentally determined a rich and accurate dataset of steady-state profiles of selected downstream kinases of PDGF-receptor-alpha mutants in combination with inhibitor treatments. Applying the tool *optPBN*, we fitted a literature-derived candidate network model to the training dataset consisting of single perturbation conditions. Model analysis suggested several important crosstalk interactions. The validity of these predictions was further investigated experimentally pointing to relevant ongoing crosstalk from PI3K to MAPK signalling in tumour cells. The refined model was evaluated with a validation dataset comprising multiple perturbation conditions. The model thereby showed excellent performance allowing to quantitatively predict the combinatorial responses from the individual treatment results in this cancer setting. The established *optPBN* pipeline is also widely applicable to gain a better understanding of other signalling networks at steady-state in a context-specific fashion.

## Introduction

Signal transduction networks are one of the core functional layers in cells. They convey intra- and extra-cellular signals towards regulators which modulate the expression of cellular phenotypes corresponding to the types and concentrations of the stimuli [1]. In general, a signal transduction network is a large and highly complex network comprising multiple intracellular signalling pathways such as mitogen-activated protein kinases (MAPK), phosphatidyl-inositide-3-kinases/AKT/mammalian-target-of-Rapamycin (PI3K/AKT/mTOR), and phospholipase C gamma/protein kinase C (PLCγ/PKC) pathways [2]. Also, there exist a number of crosstalk interactions between these signalling pathways which help to fine-tune signals and to preserve the whole networks’ integrity upon perturbations [3–5]. In a physiological condition, signals from various stimuli are integrated and transduced to regulate cellular functions and to maintain homeostasis of cellular components [6]. Once the transfer processes of these signals are deregulated, the resulting aberrant signals often lead to abnormalities of cellular functions which were identified as the etiology of many diseases including neurodegenerative diseases, metabolic diseases, as well as cancers [7–9].

Gastrointestinal stromal tumours (GISTs) are the most common primary mesenchymal neoplasia of the gastrointestinal tract. These tumours commonly arise from gain of function mutations of type III receptor tyrosine kinases, i.e. KIT in 78–90% of the cases and platelet-derived growth factor alpha (PDGFRα) in 5–7% of the cases [10]. As constitutively active surface receptors, these mutated proteins are the most upstream components of the cellular signal transduction network. The first-line therapy for GISTs is surgical resection combined with administration of Imatinib mesylate, a tyrosine kinase inhibitor with activities against ABL, BCR-ABL, KIT, and PDGFRα/β. The clinical outcome of the first-line therapy is 35%-49% 9 years survival [11]. However, certain point mutations in GIST, e.g. an Aspartate (D) to Valine (V) mutation at amino acid 842 on the *PDGFRA* gene, were also shown to be associated with drug resistance [12]. Several hypotheses were proposed to explain the underlying molecular mechanisms of oncogenic PDGFRα-driven GIST formation such as the retention of oncogenic PDGFRα in intracellular compartments [13] or the deregulation of downstream oncogenic signalling pathways, see also [14]. Such hypotheses still require further investigation at the molecular level to understand how signals are transduced and processed mechanistically in this cancer setting.

In recent years, various modelling approaches in Systems Biology were applied to model and analyse the properties of signal transduction networks in both physiological and pathological conditions. This includes Bayesian networks [15], Boolean networks (BNs) [16], fuzzy logic models [17], ordinary differential equation (ODE)-based models [18], partial differential equation (PDE)-based models [19] and also stochastic models [20], to list only a few examples. Some modelling work also demonstrated the connections between the deregulation of signal transduction networks to the pathophysiology of diseases, e.g. in metabolic disease [21] and in cancers [22]. The choice of a suitable modelling framework is depended on the research questions and the associated applications.

In our study, we aim to assess the relevance of proposed crosstalk interactions from literature in the context of deregulated platelet derived growth factor (PDGF) signalling in gastrointestinal stromal tumour (GIST) based on steady-state protein data. On the experimental part, we investigated a combination of different PDGFRα mutants and signalling molecule inhibitors to dissect the molecular structure of the deregulated PDGF signalling pathway and to generate an extensive corresponding set of steady-state protein data. On the modelling part, we applied probabilistic Boolean network (PBN) modelling, originally introduced by Shmulevich et al. to model gene regulatory networks in the context of uncertainty [23], to assess the crosstalk relevancy in PDGF signalling. With the assignment of probabilities on Boolean interactions, a binarised-state PBN is capable to depict individual regulatory effects encoded as Boolean rules in a stochastic manner and is thus suitable for determining the uncertain relevance of crosstalk interactions. In addition, the molecular activities at steady-state can also be represented by the steady-state distribution in the PBN framework, allowing them to be directly compared to the normalised (non-discretized) steady-state protein data. For a recent review see [24].

Apart from PBNs, there also exist many other modelling frameworks for the study of signal transduction networks. However, each of them has some drawbacks when applied to this particular case study. Describing the connections between signalling molecules with conditional probabilities in a Bayesian networks framework express their relationships quantitatively, but such values do not capture the underlying regulatory mechanisms between molecules which can be simplified and encoded as Boolean rules. Boolean networks can depict regulatory mechanisms of biochemical interactions with logic operators. However, the results do not capture finer scale quantitative details of molecular kinetics. The variant many-valued logic networks allow for a more fine-tuned description of molecular states but still suffer from the inherent deterministic nature and does not allow to capture the uncertainty of network interactions. Extensions of Boolean networks, e.g. by adding uncertainty in network states (e.g. by assigning random initial conditions and/or random sequences of inputs) and accumulating the stochastic results from multiple runs could also be applied to derive quantitative measures from the same system [6,25]. However, one has to explicitly impose these additional considerations on top of the conventional Boolean network framework while the probabilistic feature is already integrated within the original PBN approach. More quantitative approaches such as fuzzy logic or ODE-based models provide additional mechanistic details of the networks on a continuous scale. However, they require an extensive amount of prior knowledge to define a suitable mathematical formula and require an extensive set of experimental data in order to infer kinetic parameters.

With respect to PBN which belongs to the group of probabilistic logic models, other related modelling frameworks in the same group such as Dynamic Bayesian networks (DBNs) and Markov Login networks (MLNs) were also described. It was shown that the analytical results from DBNs usually give similar results as PBN once the state transitional diagram is established [26]. However, non-linear behaviour in biological network such as complex formation could not be directly represented by conditional probabilities in dynamic Bayesian network while such interaction can simply be encoded with logical operators in the PBN framework. In addition, it was shown that PBNs can also be represented in a more generalised form as Markov Logic networks (MLNs) [27]. This approach is often applied in genetic studies where the network structure is largely unknown and the connectivity between network components could be highly dense [28]. Nevertheless, MLNs might not be suitable for the study of signal transduction networks in our case as the connections between molecules often occur in a one-to-one or one-to-several manner and directional relationships between signalling molecules are mostly well-documented. We therefore chose and applied PBN in our case study where we could show that the PBN model is applicable for analysing the relevance of crosstalk interaction. In addition, we demonstrated that the PBN model could also predict combinatorial treatment results from individual stimulation measurements with high accuracy.

## Materials and Methods

### Biological materials

The PDGFRα-mutant proteins were generated based on the pLNCX2-PDGFRα wild-type expression plasmid generously provided by Prof. Andrius Kazlauskas (Boston). This sequence was cloned to pcDNA5/FRT/TO-vector (Invitrogen™) and the constitutively active oncogenic PDGFRα mutant occurring in GIST was generated by introducing the D842V point mutation (PDGFRα-D842V-wild type, *“DV-WT”*). Based on the PDGFRα-D842V mutant, two PDGFRα-D842V-*”knock-out”* mutants were constructed by introducing tyrosine (Y) to phenylalanine (F) point mutations, i.e. Y720F (PDGFRα-D842V-Y720F, *“DV-dMAPK”*) and YY731/742FF (PDGFRα-D842V-YY731/742FF, *“DV-dPI3K”*) which have been shown to abrogate the recruitment of signalling molecules such as SHP2 and PI3K in the PDGFRα-wild type receptor, respectively. All point mutations were introduced using the QuikChange kit (Stratagene) following the manufacturer’s recommendations.

For analysing the signalling behaviour of the mutant PDGFRα protein, an isogenic Flp-In™ cell line was constructed based on the Hek293 cell line which is naturally devoid of endogenous expression of PDGFRα/β proteins. As such, Hek293 cells were co-transfected with the Flp-In™ target site vector (pFRT/lacZeo, Invitrogen™) and the regulatory vector (encoding Tetracycline repressor protein, pcDNA™6/TR/invitrogen) using the TransIT®-LT1 transfection reagent (Mirus) according to the manufacturer’s recommendations. Cell clones were selected by cultivation in presence of 10μg/ml Blasticidin and 100μg/ml Zeocin™ (both InvivoGen). Based on this isogenic parental cell line (“293FR”), stable cell lines were generated by site directed recombination by co-transfecting the transgene expression plasmid (pcDNA5/FRT/TO-based) in combination with the Flp recombinase expression plasmid (pOG44, Invitogen). Stably transfected cells were selected and cultivated in presence of 100μg/ml Hygromycin and 10μg/ml Blasticidin [13].

### Cell treatment and Western blot analysis

Experiments were conducted by seeding 250,000 cells/well (12 well plate) in DMEM containing 10% FBS, 2% L-Glutamine, and 25mM HEPES on 12-well plates for 24–30 hours. Then, growth media was exchanged and protein expression was induced by adding 5ng/ml doxycycline (Sigma) under serum reduced (1% FBS) conditions for 14 hours and for additional 3 hours under serum free (0% FBS) conditions. Pharmacological inhibition was performed by adding either 1μM of the PI3K inhibitor Wortmannin, 10μM of the MEK1,2 inhibitor U0126, 1μM of the pan-PKC inhibitor GF109203X, or 500nM for the PKC-α and PKC-β1 specific inhibitor Gö6976. Wortmannin and Gö6976 were purchased from Sigma-aldrich while GF109203X and U0126 were purchased from Calbiochem.

Cells were lysed on the dish with 300μl 1x Lämmli buffer. Cellular proteins were subjected to SDS-PAGE, transferred to a nylon membrane (Amersham Hybond™-N/GE Healthcare by ThermoFisher Scientific), blocked with 10% BSA and probed with the respective antibodies. Alpha-tubulin was also probed as loading control. Phospho-specific antibodies against ERK1/2 (pThr202/pTyr204), PDGFRα (pTyr849)/β(pTyr857), AKT (pSer473) and PKC substrates (pSer) were purchased from Cell Signaling. Phospho-specific antibodies for STAT5 (pTyr694) and PLCγ1 (pTyr783) were purchased from BD Biosciences. Antibodies against PDGFRα (C-20) were purchased from Santa Cruz Biotechnology®. Each gel was probed simultaneously against alpha-tubulin to allow compensation for loading variations (using either antibody DM1A/Santa Cruz Biotechnology® or PA1-38814/Pierce™). The secondary antibodies were coupled with IRdye, allowing imaging with the LI-COR Odyssey system. The relative intensities of Western blot images were quantified with Image Studio Lite version 4.0 using left-right and top-bottom background subtraction. The experiments were done in 3 biological replicates with 3 technical replicates on each Western blot. The calibrator sample was prepared from an early passage of DV-WT cell line induced by 5ng/ml doxycycline.

### Normalisation pipeline and datasets for modelling

Starting from the obtained raw experimental data, a quality control step was applied by discarding 1) the data points overlapping with unspecific stains and 2) the data points with low signals due to blotting issues. Furthermore, data points with a corresponding tubulin signal of less than 20 percent compared to the maximal signal within the same blot were removed from the analysis due to low signal to noise ratio.

The remaining data points were first normalised to tubulin (loading control) and then to a calibrator sample in order to correct for the differences across multiple blots. The normalised mean of technical triplicates from each biological replicate were pooled and re-normalised to the maximal value to generate the final mean and standard deviation for the modelling task.

We divided the initial set of experimental data into two parts. The training dataset comprises 6 experimental conditions including negative control (all signals assumed to be zero), positive control (DV-WT) and 4 experimental conditions with single perturbations by YF point mutations (DV-dMAPK and DV-dPI3K) or signalling inhibitors (DV-WT-Wortmannin and DV-WT-U0126). The validation dataset comprises the 4 remaining experimental conditions with combined perturbations.

### Literature-derived PDGF signalling network

We built a mutant PDGF signalling network including the major downstream signalling pathways including MAPK, PI3K/AKT/mTOR, PLCγ/PKC pathways, as well as STAT5 as another prominent signalling target downstream of the oncogenic mutant (but not downstream of the wild-type receptor) [13]. Interactions and regulatory mechanisms were modelled according to well-established knowledge [29,30]. The recruitment sites of upstream signalling molecules to PDGFRα were previously described [31,32]. Among the three major downstream signalling pathways in PDGF signalling, there also exist a number of crosstalk interactions suggested by the literature as listed in Table 1. In addition, apart from depicting the effects of inhibitors on their main targets, we also included the information on an off-target effect of Wortmannin on conventional PKC [33] which might be essential to explain the obtained signalling profiles in the context of GIST.

**Table 1:** References for the investigated crosstalk interactions in the PDGF signalling network.

Crosstalk number	Descriptions	References
1	Ras -> PI3K	[34]
2	MEK1,2 -| Grb2SOS	[35]
3	PI3K -> Ras	[3]
4	PI3K -> MEK1,2	[5]
5	PIP3 -> GabSOS	[36]
6	Akt -| GabSOS	[37]
7	Akt -| Raf	[4]
8	PLCγ -> PI3K	[38]
9	PKC -> MEK1,2	[5]

The crosstalk interactions among MAPK, PI3K/AKT/mTOR and PLCγ/PKC pathways within PDGF signalling as suggested by literature are listed. The annotation ‘->‘ refers to activation and the annotation ‘-|’ refers to inhibition.

### PBN description of PDGF signalling network

Based on the topology of the literature-derived PDGF signalling network, we built a corresponding PBN model which comprises 27 nodes (molecules) and 40 edges (interactions). Multiple interactions directing onto one node were modelled as separated Boolean rules with the corresponding selection probabilities. We applied the Boolean logic gate “OR” to combine non-exclusive inputs in the same class while inhibitions were represented by the combined “AND” and “NOT” gates. Additionally, selection probabilities of the Boolean rules that represent the main pathways were assigned to be high with the flag “H”, while being fixed to low with the flag “L” for all crosstalk interactions. This assignment ensures that the optimised selection probabilities for the interaction(s) with the flag “H” will always be higher compared to the interaction(s) with the flag “L”. For instance, if there are two activating interactions directing towards a single target node while one being from the main pathway and another being from the crosstalk interaction, the bound of the optimised selection probability for the main interaction (with flag “H”) will be from 0.5 to 1.0 while it will be from 0 to 0.5 for the crosstalk interaction (with flag “L”). This implementation is integrated in the latest version of the *optPBN* toolbox (version 2.2.3) available on http://sourceforge.net/projects/optpbn. The full set of model descriptions and the implementation of the Boolean rules including the assignment of “H” and “L” flags can be found in S1 File and the computational scripts of the PBN models, as well as the example of results are included in S2 File.

### Optimisation

**Fig 1:**
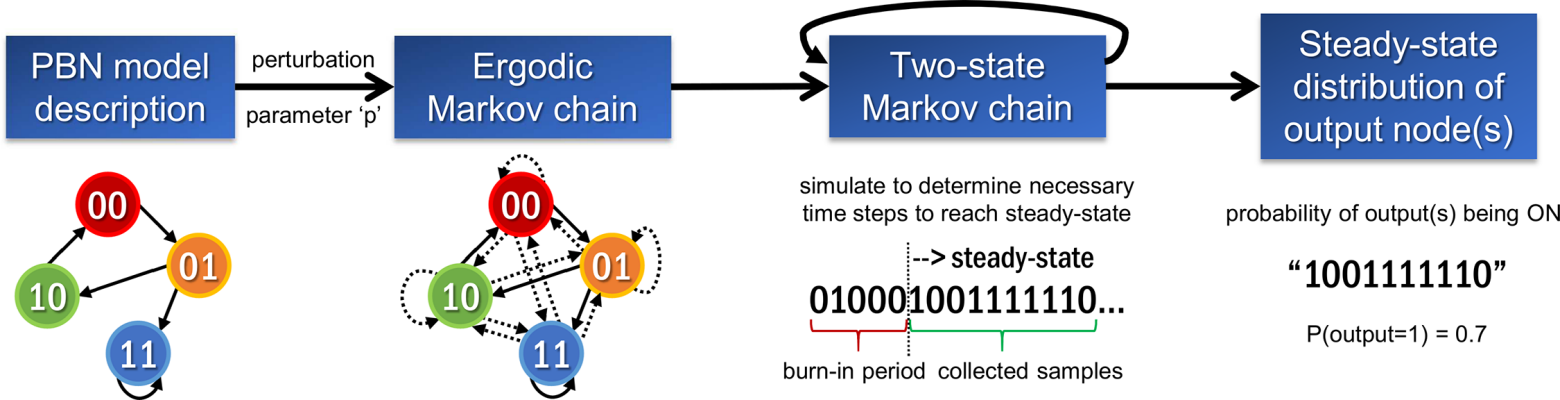
Approximation of the steady-state distribution in the *optPBN* framework. The dynamic behaviour of the PBN model was represented as a Markov chain. A small perturbation parameter ‘p’ was introduced to ensure the ergodicity of the respective Markov chain which is rendered *irreducible* (all states can be reached by any other states) and *aperiodic* (all states can be revisited in a non-periodic manner) thus possessing a unique steady-state distribution independent of the initial conditions. The two-state Markov chain approach was subsequently applied to determine the number of necessary time steps to reach the steady-state (burn-in period) and to collect sufficiently large number of samples in order to approximate the marginalised steady-state distribution of the output states for a defined (adjustable) accuracy. Note that multiple evaluations of the burn-in period and collected samples might be needed. The approximated steady-state distribution corresponds to the probability of the output node(s) being ON, i.e. being 1, as determined from the collected samples.

We applied the grid-based version of the *optPBN* toolbox (version 2.2.3) to perform the optimisation and analysis [39]. The model description of the PDGF signalling network in the PBN format was combined with the steady-state measurement data of 6 signalling molecules including the phosphorylated forms of PDGFRα, of PLCγ, of STAT5, of PKC substrates, of ERK1,2, and of AKT, to generate an optimisation problem. The aim of the optimisation is to identify the selection probabilities of each Boolean interaction in the PBN model that return steady-state properties, i.e. the stationary distributions of the molecular states, which match the measurement data. In the *optPBN* framework, we represent the dynamics of a PBN as an ergodic Markov chain where we ensure ergodicity by introducing a small perturbation parameter ‘p’ to randomly perturb the states [40]. The resulting ergodic Markov chain is therefore *irreducible* (all states can be reached by any other states) and *aperiodic* (all states can be revisited in a non-periodic manner) thus possessing a unique stationary distribution regardless to the initial conditions. The two-state Markov chain approach was subsequently applied to determine the number of necessary time steps to reach steady-state and to approximate the marginalised steady-state distribution of each output state with a certain accuracy [41] (see also the pipeline in Fig 1). All parameter values were chosen according to the previous study [39] i.e. the perturbation parameter ‘p’ = 0.001, the range of accuracy parameter ‘r’ = 0.025, and the probability to acquire results at the defined accuracy ‘s’ = 0.95, to ensure the creation of ergodic Markov chains based on random perturbations while the steady-state distribution of each node was minimally perturbed and the approximation of the steady-state distribution is relatively accurate. In the last step, the sum of squared error (SSE) comparing the simulated molecular states and the measurement data was calculated as indicator for the goodness-of-fit.

In the investigated PBN model of PDGF signalling, up to 27 selection probabilities were optimised on the Grid’5000 infrastructure using 160 parallel cores (Intel CPU @2.50Ghz, 16GB ram). 5,000 samples of parameter sets were evaluated by two optimisation algorithms, namely, differential evolution (DE) and evolutionary algorithm (EA). Three rounds of optimisation were performed where we observed that the optimisation results generated by the DE algorithm were converged already after 1,500 iterations (result not shown). The best fitting cost from the DE algorithm was chosen for the comparison of the fitting quality between model variants along with Akaike’s Information Criterion (AIC) [42,43] and the F-test for regression model. The mean and distribution of the optimised selection probabilities based on the best 500 parameter sets were subsequently analysed.

### Modelling of the deregulated PDGF signalling in BN and ODE-based frameworks

We applied the *BN/PBN toolbox* [44] and the *Systems Biology toolbox 2 (SBTB2)* revision 72 [45] to model and analyse the deregulated PDGF signalling in GIST also in BN and ODE frameworks, respectively. Regarding modelling in the BN framework, we removed all negative feedbacks on the node PDGFR, removed the basal activities of PTEN and PDK, as well as assumed that Wortmannin does not inhibit PKC efficiently, in order to obtain the best possible fitting in the BN framework. For ODE modelling, we applied the law of mass action to convert the interaction graph of deregulated PDGF signalling into a set of ordinary differential equations, see an example in [20]. We also added deactivation/degradation reactions and their corresponding parameters for each molecule to balance the activation/synthesis. 20 independent optimisation runs of the ODE model were performed using 50,000 parameter sets applying the particle swarm algorithm (global optimiser) followed by testing another 50,000 parameter sets applying the Simplex algorithm (local optimiser) with a parameter range from 0 to 100. The best fitting cost from the ODE model was compared to the ones from the BN and PBN approaches while the distribution of optimised parameters from all 20 optimisation runs was compared to the ones of the PBN model. The complete set of computational scripts and modelling results is available on http://sourceforge.net/projects/optpbn.

## Results

### Incomplete inhibition and potential influence of crosstalk interactions were observed from experimental data

**Fig 2:**
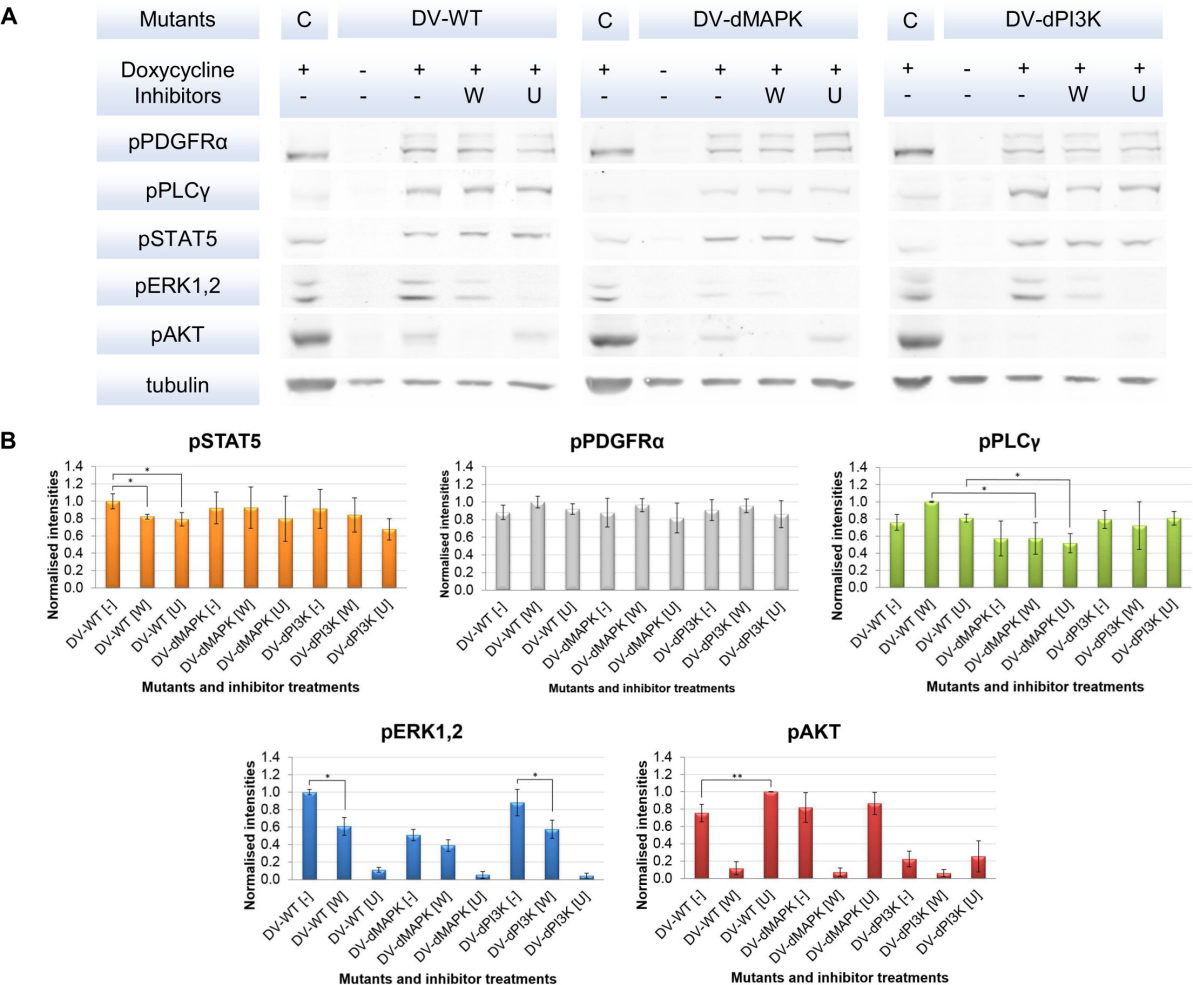
Raw and quantified data from the initial set of Western blot experiments. [A] An example set of raw data from Western blot experiments is shown in Fig 2A. Three PDGFRα mutants containing the D842V point mutation were investigated. One mutant contains no additional YF point mutation, i.e. wild type (DV-WT) while the other two contain either Y720F (DV-dMAPK) or YY731/742FF (DV-dPI3K) point mutations which abrogate the recruitment sites of upstream signalling molecule for MAPK and PI3K/AKT/mTOR pathways, respectively. These experimental conditions were combined with the treatment with two signalling inhibitors, i.e. Wortmannin (W) [1 μM], which mainly inhibits PI3K, and U0126 (U) [10 μM], which mainly inhibits MEK1,2. Doxycycline is used to induce the transcription of the constitutively phosphorylated PDGFRα mutants. The experiment was performed in 3 biological replicates with 3 technical replicates. The signals from the calibrator sample (C) derived from the induced DV-WT cell line in an early passage were applied to calibrate relative intensities between Western blots. [B] The summarised quantified Western blot data are shown in Fig 2B. The signals were normalised against tubulin and subsequently calibrated by the calibrator signals. The normalised mean of technical triplicates from each biological replicate were pooled and re-normalised to the maximal value to generate the final mean and standard deviation values for modelling task. The significances between two data points were assessed with the Student’s T-test; p-value < 0.05 (*) and p-value < 0.01 (**).

We investigated the signalling profiles of three PDGFRα mutants, i.e. DV-WT, DV-dMAPK and DV-dPI3K in combinatorial treatment with two signalling inhibitors, i.e. Wortmannin and U0126. According to the results from the Western blot investigation (Fig 2), the cell system and the signalling inhibitors functioned properly. We observed that PDGFRα transgenes were expressed exclusively upon doxycycline induction and that basal phosphorylation of the signalling molecules was very low. Also, phosphorylated PDGFRα (pPDGFRα) signals were found to be comparable between the different mutants (Fig 2). This observation also applies to phosphorylated STAT5 (pSTAT5) signals. The phosphorylated PLCγ (pPLCγ) signals dropped in the DV-dMAPK mutant, indicating that the SHP-2 recruiting motif Y720 also affects PLCγ activation (see Fig 2). In parallel, decreased activities of downstream signalling molecules, i.e. phosphorylated ERK1,2 (pERK1,2) and phosphorylated AKT (pAKT) were observed in accordance to the inhibitory effects of YF mutants and the signalling inhibitors. These results indicate that the abrogation of recruitment sites by point mutations and the signalling inhibitors worked effectively on their primary targets. In addition, we found that neither the constructions of our cell system nor the inhibitor treatments used in the study affect the expression level of the investigated signalling components (see [13] and S1 Fig).

Among the inhibitory effects that we investigated, we observed that both Wortmannin and U0126 reduced the phosphorylation of their downstream signalling targets almost completely. In contrast, the DV mutants with additional YF point mutations delivered only partial inhibition, e.g. the signals of pERK1,2 and pPLCγ in DV-dMAPK mutants are only reduced by half. In parallel, some evidences of crosstalk interaction were observed in the dataset. For instance, inhibiting PI3K with Wortmannin also decreased the pERK1,2 signal in the MAPK pathway while inhibiting MEK1,2 with U0126 additionally increased the pAKT signal in the PI3K/AKT/mTOR pathway (Fig 2B). It should be noted that the cross-regulation through Wortmannin inhibition is stronger than the one via U0126, with up to 39% decreased pERK1,2 signal versus up to 25% increased pAKT signal compared to the untreated conditions. Also, the cross-regulation by Wortmannin is significant in 2 out of 3 mutants while the crossed inhibitory effect mediated by U0126 was significant only in the DV-WT condition (Fig 2).

### Initial data integration in the PBN framework suggested important crosstalk interactions from candidate network

**Fig 3:**
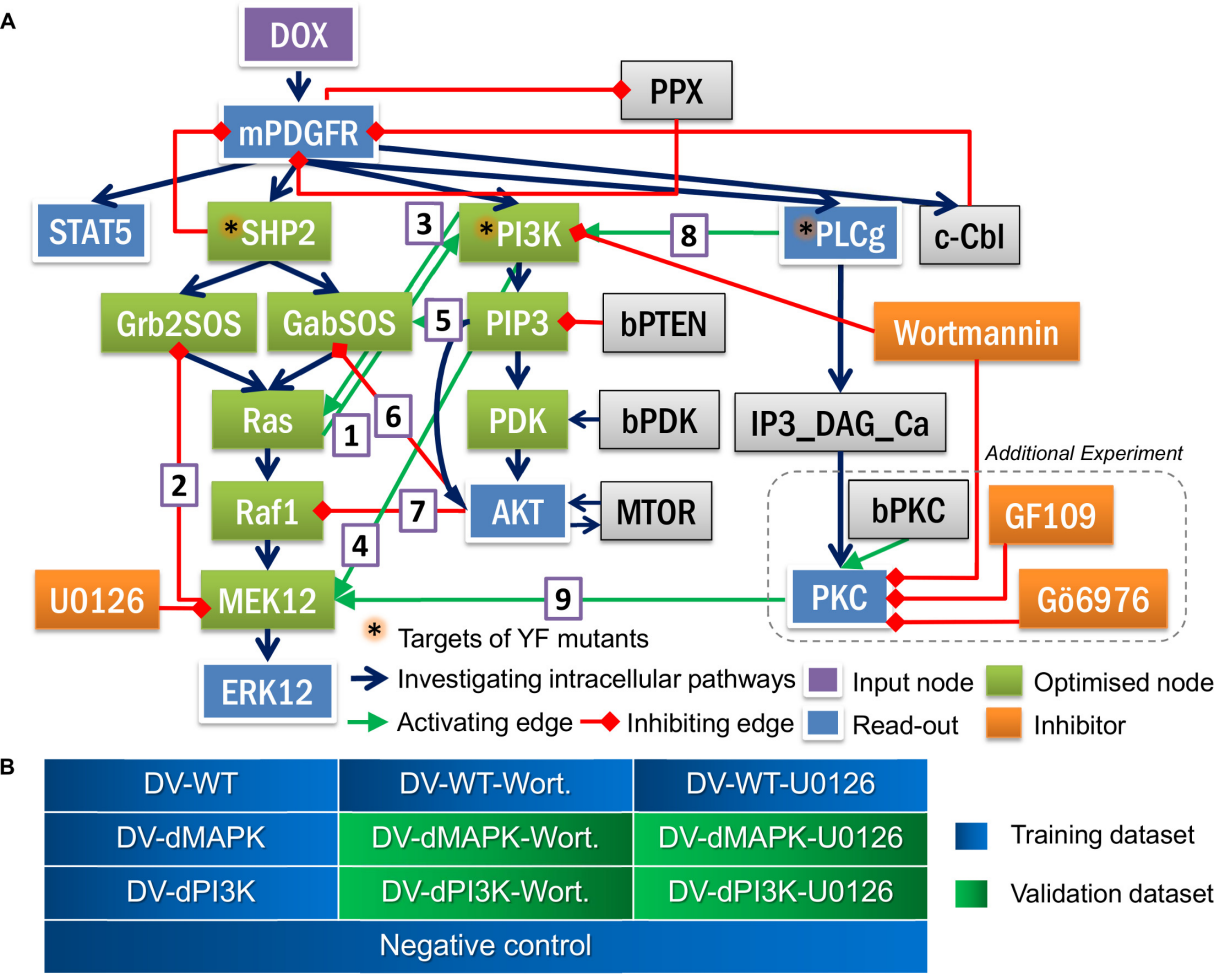
Model structure and datasets for modelling. **[A]** The model structure of the literature-derived PDGF signalling is shown in Fig 3A. The constitutive activity of mutated PDGFRα (mPDGFR) is inducible by doxycycline (DOX). Downstream of mPDGFR includes 3 canonical intracellular signalling pathways: MAPK, PI3K/AKT/mTOR and PLCγ/PKC pathways as well as regulatory mechanisms on PDGFRα. In addition, we included, STAT5, which was shown to be rather activated by oncogenic PDGFRα mutants [13]. SHP2, PI3K and PLCγ, marked with asterisks, are the targets of YF mutants. The basal activities of PTEN and PDK were represented by bPTEN and bPDK, respectively. Four signalling inhibitors, i.e. Wortmannin, U0126, GF109231X (GF109), and Gö6976, and their targets are also depicted. The phosphorylation state of five downstream signalling molecules, i.e. PDGFRα, STAT5, PLCγ, ERK1,2 and AKT were used as read-outs. There were 9 crosstalk interactions proposed in literature (numbered 1–9) that might be involved in PDGF signalling. Note that the phosphorylation of PKC substrates (PKC) was also included as a read-out in the additional experiment (Fig 4) and the read-out nodes with multiple inputs, i.e. PDGFRα, PLCγ, PKC and AKT are also optimised. **[B]** The quantified Western blot data from 9 experimentally-investigated conditions together with a negative control condition were applied for the modelling task. Experimental data were split into a training set (single perturbation plus positive and negative controls) and a validation dataset (combinatorial perturbations).

We integrated this initial dataset into the literature-derived PBN model of PDGF signalling by applying the tool *optPBN*. The model structure and the splitting of datasets are shown in Fig 3. Initially, we started with a model topology without any crosstalk interaction to explore if the model that contains only the major oncogenic pathways would already be sufficient to fit the training dataset. The results showed that the initial model fitted well to pPDGFR, pSTAT5 and pAKT data, but still could not capture the decrease of pERK1/2 signals after Wortmannin treatment. Also, we found that a model variant with all-or-none inhibition could not fit well to either the pERK1/2 or the pPLCγ data (see S2 Fig).

Next, we investigated 9 model variants where the crosstalk interactions proposed in the literature (Table 1) were added one-at-a-time and optimisation was re-performed (Table 2).

**Table 2:** Fitting costs and AIC value of the initial model variants with and without crosstalk interactions.

Crosstalk number	Descriptions	Fitting cost	AIC value
-	No crosstalk	0.198	-104.62
1	Ras -> PI3K	0.193	-101.39
2	MEK1,2 -| Grb2SOS	0.197	-100.77
**3**	**PI3K -> Ras**	**0.155**	**-107.97**
**4**	**PI3K -> MEK1,2**	**0.147**	**-109.56**
5	PIP3 -> GabSOS	0.191	-101.70
6	Akt -| GabSOS	0.190	-101.86
7	Akt -| Raf	0.194	-101.23
8	PLCγ -> PI3K	0.197	-100.77
**9**	**PKC -> MEK1,2**	**0.130**	**-113.24**

The fitting cost and AIC value of the initial PBN model without any crosstalk interaction was compared to the ones after adding crosstalk interactions one-at-a-time. The fitting cost is the sum of squared error between the simulated values and the mean values of measurement data in 6 experimental conditions within the training dataset. The annotation ‘->‘ refers to activation and the annotation ‘-|’ refers to inhibition.

Only the positive crosstalk interactions from PI3K to Ras (Number 3), from PI3K to MEK1,2 (Number 4) and from PKC to MEK1,2 (Number 9) could further improve model fitting and allowed to distinguish the signal intensities of the DV-WT and DV-WT-Wortmannin conditions (results not shown). In addition, the lowest AIC values and highest F values of these model variants also support that these are the most preferential models to be further investigated.

### Iterative experimental investigation and model refinement revealed that PKC activity is independent of PDGFRα activation and the crosstalk interactions from PI3K towards MAPK pathway being the most relevant

**Fig 4:**
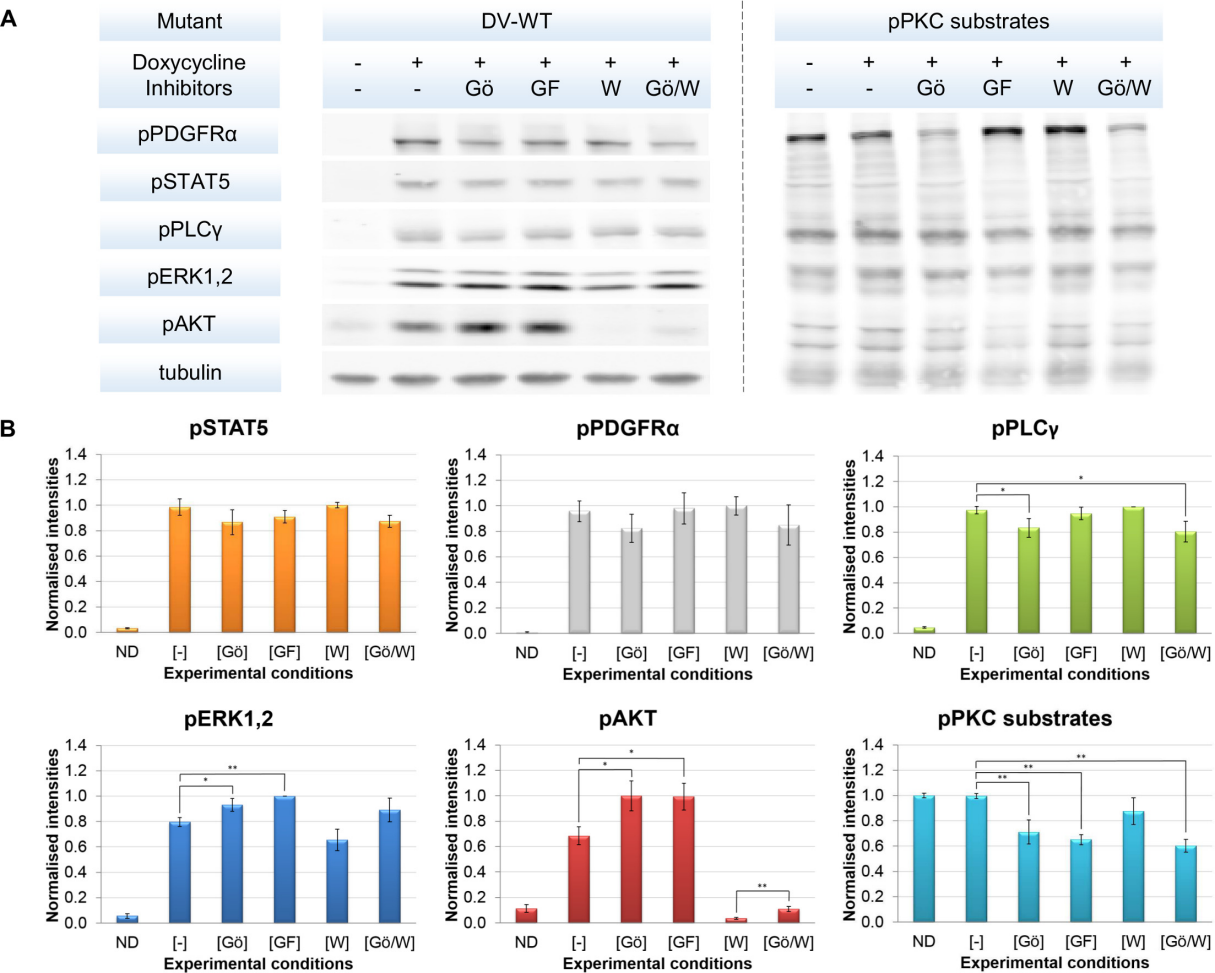
Raw and quantified data from the additional set of Western blot experiments. **[A]** An example set of raw data from the additional Western blot experiment to investigate the validity of modelling results are shown in Fig 4A. The PDGFRα mutant with D842V point mutation (DV-WT) was investigated. Two additional signalling inhibitors, i.e. PKC-α and PKC-β1 specific inhibitor Gö6976 and pan-PKC inhibitor GF109203X together with the detection of phosphorylated PKC (pPKC) substrates were included. Six experimental conditions were assigned, i.e. negative control without doxycycline induction (ND), positive control with doxycycline induction but without inhibitor treatment (-), doxycycline induction with the treatment of Gö6976 (Gö) [500nM], of GF109203X (GF) [1μM], of Wortmannin (W) [1μM] and of combined Gö6976 and Wortmannin (Gö/W) [500nM/1μM]. The experiment was performed in 3 biological replicates with 3 technical replicates. **[B]** The summarised quantified data from the additional Western blot experiment are shown in Fig 4B. Data were normalised against tubulin, then the mean values of technical triplicates from each biological replicates were pooled and re-normalised to maximal values. The significances between two data points were assessed with the Student’s T-test; p-value < 0.05 (*) and p-value < 0.01 (**).

To investigate the validity of these modelling results, we set-up an additional experiment by treating the DV-WT mutant with PKC inhibitors (Pan-PKC inhibitor ‘GF109231X’ or conventional-PKC specific inhibitor ‘Gö6976’) thereby also probing for phosphorylated PKC (pPKC) substrates to investigate if the PKC inhibitors worked effectively (Fig 4).

While the PKC inhibitors exhibited a strong effect on their downstream primary target, the results showed that PKC activity is independent of PDGFRα activation. Considering the positive crosstalk interaction from PKC towards MEK1,2, we initially expected to observe decreased activities of pERK1,2 upon the reduction of pPKC substrate signals. However, we witnessed to the contrary that the pERK1,2 signals increased after PKC-inhibitors treatments. Regarding the fact that our initial model assumed pPKC signals to be dependent on pPDGFR activation, we concluded that the crosstalk interaction from PKC towards MEK1,2 is not important in our cellular context.

On the counterpart, we still observed that pERK1,2 signals were decreased upon Wortmannin treatment as previously shown in the initial set of experiments (Figs 2 and 4). This finding supported the validity of the positive crosstalk interactions from PI3K towards MAPK pathways. In addition, we saw that pAKT and pERK1,2 signals are significantly up-regulated following PKC-inhibitor treatment. On the one hand, it could be an off-target effect of the two PKC inhibitors on the negative regulators of both oncogenic pathways. On the other hand, the off-target effect might only be applied to PI3K/AKT/mTOR pathway while the changes in pERK1,2 signals were modulated by the positive crosstalk as suggested by the model.

Further model refinement by introducing basal PKC activity and by adding the 3 data points from negative control, positive control and Wortmannin treatment conditions to the training dataset confirmed in the one-at-a-time crosstalk adding experiment (Table 3, see also Table 2 for comparison) that the crosstalk interactions from PI3K to MAPK pathway are the only relevant interactions that improve model fitting. We chose the refined model with the crosstalk from PI3K to MEK1,2 which has the best fitting cost as the final model for further analysis. Additional information on model complexity, the F-test and fitting costs from 3 optimisation runs can be found in S3 File.

**Table 3:** Fitting costs and AIC value of the refined model variants with and without crosstalk interactions.

Crosstalk number	Descriptions	Fitting cost	AIC value
-	No crosstalk	0.208	-117.20
1	Ras -> PI3K	0.200	-114.50
2	MEK1,2 -| Grb2SOS	0.200	-114.50
**3**	**PI3K -> Ras**	**0.163**	**-121.25**
**4**	**PI3K -> MEK1,2**	**0.162**	**-121.45**
5	PIP3 -> GabSOS	0.200	-114.50
6	Akt -| GabSOS	0.197	-114.99
7	Akt -| Raf	0.202	-114.17
8	PLCγ -> PI3K	0.197	-114.99
9	PKC -> MEK1,2	0.201	-114.33

The fitting cost and AIC value of the refined PBN model variant without crosstalk interaction was compared to the ones after adding crosstalk interactions one-at-a-time. The fitting cost is the sum of square error between the simulated values and the mean values of measurement data in 6 experimental conditions within the training dataset. The annotation ‘->‘ refers to activation and the annotation ‘-|’ refers to inhibition.

**Fig 5:**
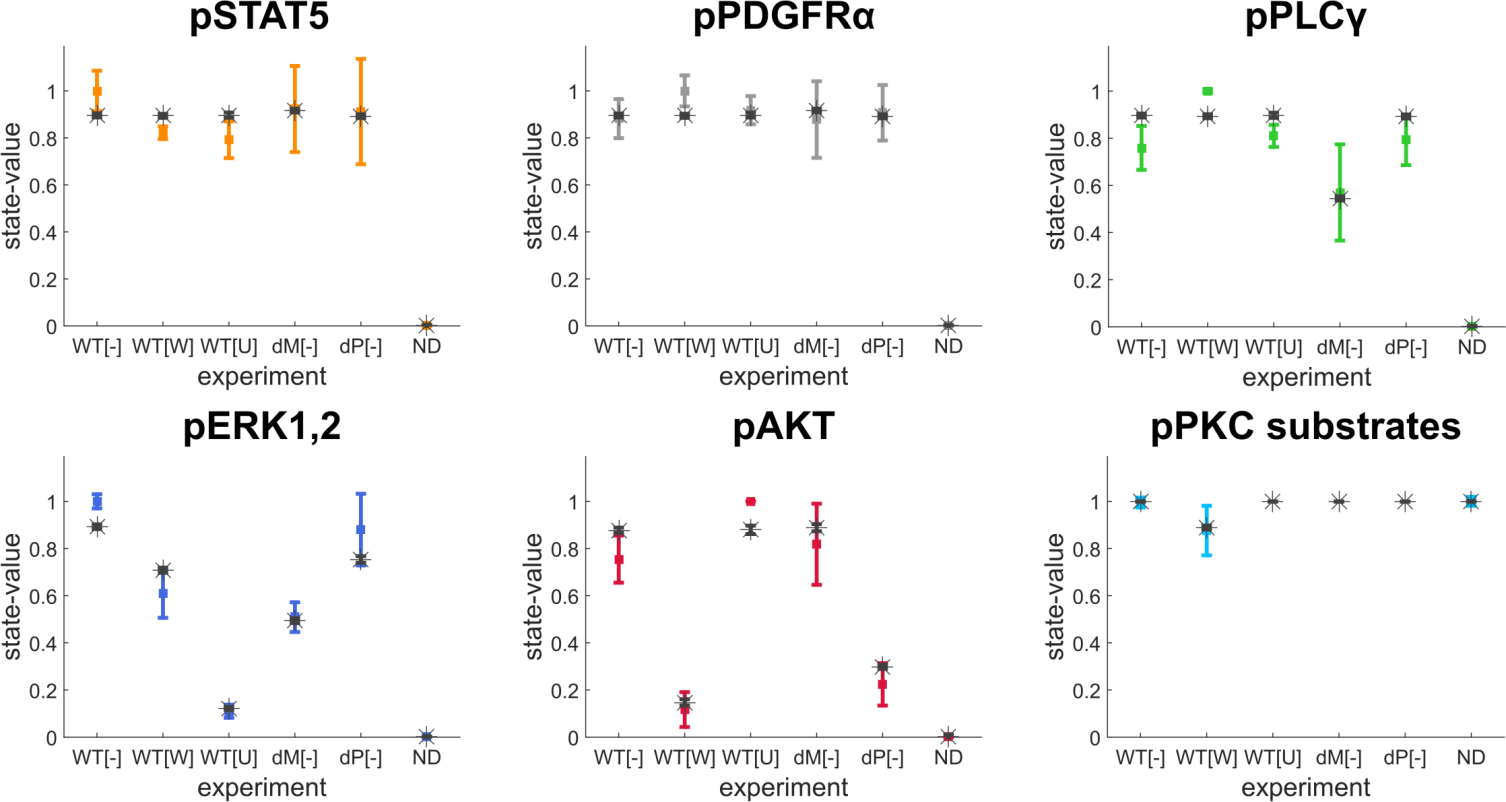
Plotted fitting results of the final PBN model. Steady-state distributions of output states were generated from the final PBN model using the *optPBN* toolbox. The mean and standard deviation (SD) of steady-state distribution from ten rounds of simulation (black stars [mean] and error bars [SD] on top) were compared against the experimental data from the training dataset (multi-coloured squares [mean] and error bars [SD] on bottom). Six experimental conditions as labelled on the x-axis are in the following order: DV-WT (WT[-]), DV-WT-Wortmannin (WT[W]), DV-WT-U0126 (WT[U]), DV-dMAPK (dM[-]), DV-dPI3K (dP[-]), and negative control (no doxycycline induction, ND).

We found that the quality of plotted fitting results generated from the final model is very good when compared to the training dataset (Fig 5) where similar results can be obtained from the model variant with the crosstalk from PI3K to Ras (results not shown). We also explored all model variants with pair-wise combinations of crosstalks and we found that only the model variants with combined crosstalks including the ones from either PI3K to MEK1,2 or from PI3K to Ras have better fitting costs. In addition, we observed that a model variant with both crosstalk interactions from PI3K to the MAPK pathway integrated could not further improve the fitting cost (see S4 File), possibly due to redundancy. Either one of these two crosstalk interactions is sufficient to explain the signalling profiles of the cancer-specific deregulated PDGF signalling in GIST.

### The final PBN model of deregulated PDGF signalling in GIST is highly predictive for combinatorial stimulations and provides quantitative insights on signal flows

**Fig 6:**
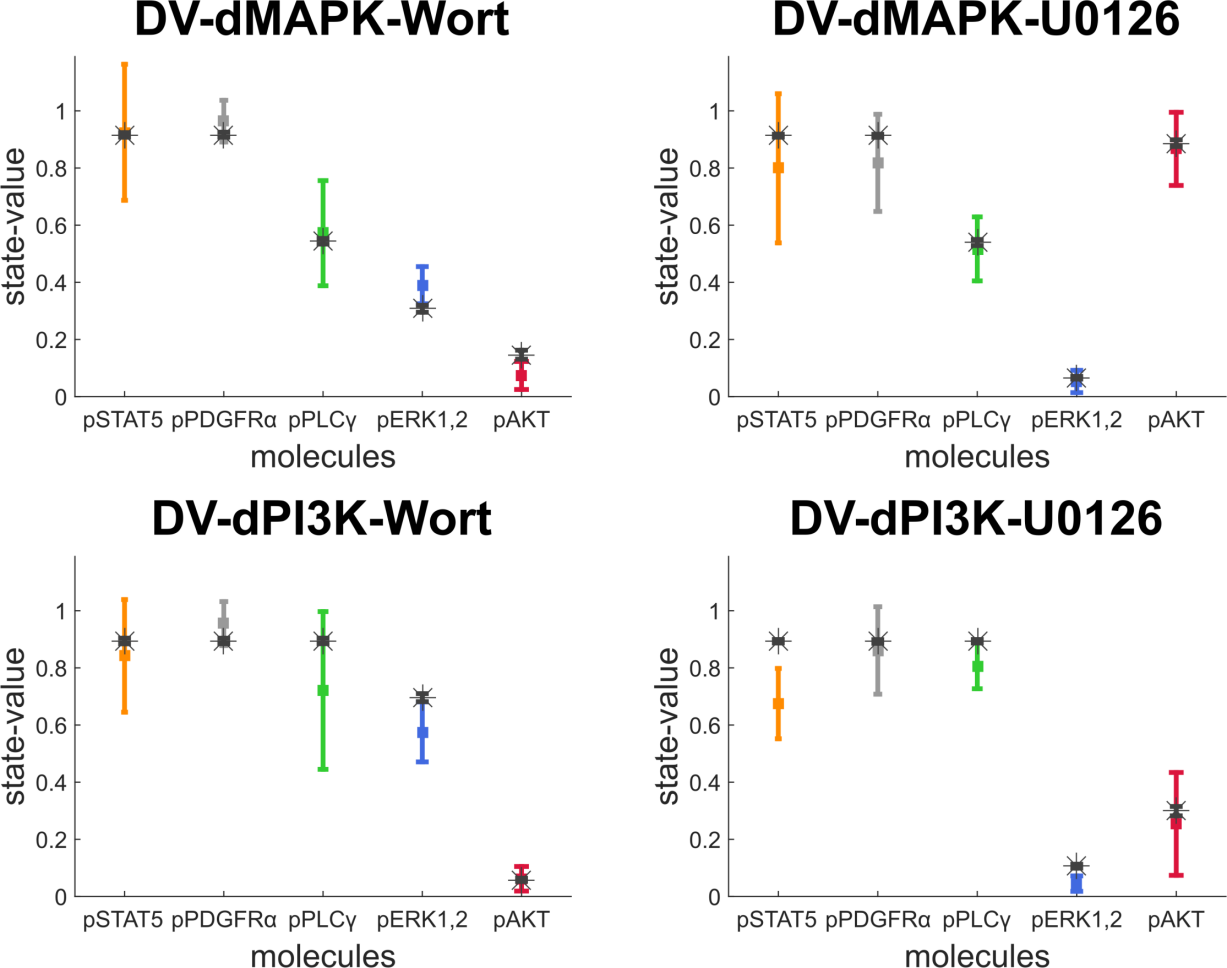
Predictions from the final PBN model compared to the validation dataset. The mean and standard deviation of ten simulated values from the final PBN model (black stars [means] and error bars [SD] on top) were compared against the experimental data from the validation dataset (multi-coloured squares [mean] and error bars [SD] on bottom). Five molecules as labelled on the x-axis in four experimental conditions are in the following order: pSTAT5, pPDGFRα, pPLCγ, pERK1,2 and pAKT.

In order to evaluate the predictive power of the final PDGF model, we simulated the model with combined perturbations conditions integrating YF point mutations (dMAPK and dPI3K) with signalling inhibitors (Wortmannin and U0126) and compared the model outputs to the respective experimental data (validation dataset). The results are shown in Fig 6.

It was shown that our final PBN model predicts the molecular profiles of the combined perturbations conditions in the validation dataset accurately. 18 out of 20 data points in combined perturbation conditions were precisely predicted within the standard deviation of the experimental data. The prediction of pERK1,2 in the DV-dPI3K-U0126 condition is close to this error range. Also, the prediction on pSTAT5 in the same condition is qualitatively captured correctly. We suspect that this data point could be an experimental outlier due to its distinctly low signal compared to the others on the same panel (see Fig 2). Overall, this analysis demonstrates the excellent performance of the final PBN model in predicting combinatorial treatments.

The model variant without any crosstalk interaction also returned a relatively good prediction, except in the experimental conditions where the necessary crosstalk interaction takes place (see S3 Fig). For instance, the discrepancy can be observed in the DV-dPI3K-Wortmannin condition where pERK1,2 signals were modulated by the crosstalk interaction from the PI3K/AKT/mTOR pathway. In addition, we found that all single perturbation experiments are needed in the training dataset in order to obtain a model with good predictive power for the combined perturbation conditions. Removal of a single perturbation experiment from the training dataset results in higher variabilities of output states (see S4 Fig).

**Fig 7:**
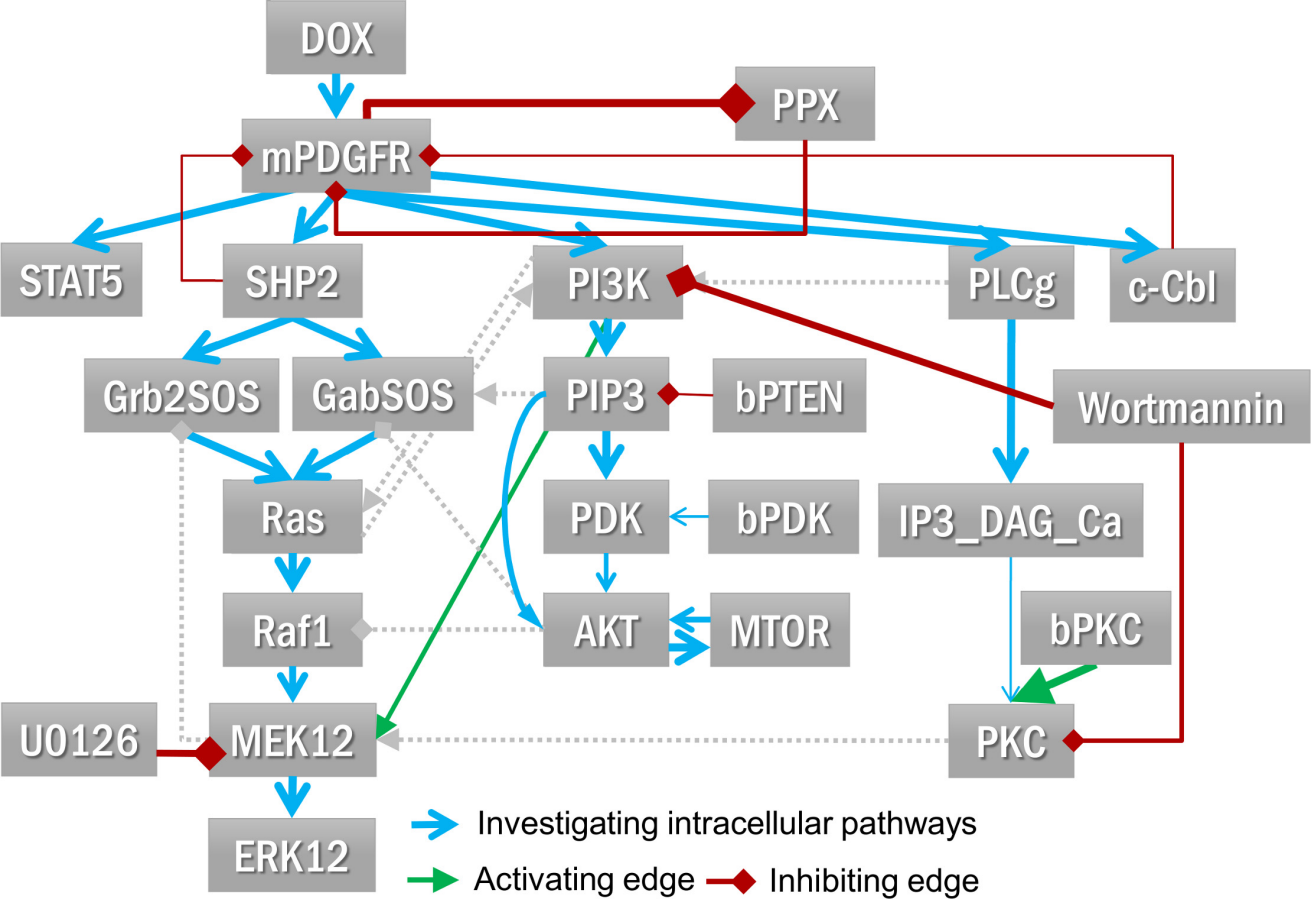
Signal flow in the final PBN model. A quantitative overview of the signal flow in the final PBN is shown. The weights of interaction as represented by the optimised selection probabilities in PBN model were classified into 5 categories: very low (< 0.1), low (0.1 to 0.3), medium (0.3 to 0.7), high (0.7 to 0.9), and very high (> 0.9) where the sizes of the arrows were adjusted accordingly. The crosstalk interactions that are not necessary in the context of GIST were replaced with dotted grey arrows.

Besides its predictive power, the final PBN model also summarises the quantitative signal flow within the deregulated PDGF signalling network in GIST by considering the weights of interactions represented by the optimised selection probabilities as shown in Fig 7.

The analysis demonstrates that among multiple crosstalk interactions that were proposed in the literature to be involved in the PDGF signalling pathway, only one crosstalk is necessary to explain the investigated experimental data in the context of GIST mutants in our cell system. Also, it is shown that the weight of the crosstalk interaction from PI3K to MEK1,2 is not very strong (0.226) compared to the main pathway (0.774). In parallel, it is shown that the weight of basal PKC activity which is independent from the PDGFRα activation completely overrides the activation signals from inositol 1,4,5-trisphosphate (IP3), diacylglycerol (DAG), and calcium ion (Ca) (0.997 versus 0.003). The known inhibitory effects of the inhibitor are also reported quantitatively, e.g. the effects of Wortmannin on its primary target PI3K (0.893) is stronger than the one on its off-target molecule PKC (0.152) while U0126 has a strong inhibitory effect on MEK1,2 (0.851) [5,46,47]. The complete list of the weights of the interactions from the final PBN model can be found in S5 File.

## Discussion

### Advantages of applying the PBN approach for analysing signal transduction networks

In this research article, we demonstrated several advantages of applying the PBN approach for analysing signal transduction networks with steady-state data. First, we experimentally showed that the inhibitory effects of biological interventions do not work in an all-or-none fashion. Such data call for modelling approaches that can capture quantitative changes of signalling molecules where PBN approach serves the purpose well for capturing quantitative changes at steady-state. Second, we showed that a PBN model with a compact model description and a minimal parameterisation is sufficient to explain the signalling profiles observed at steady-state. For a comparative purpose, an additional study on the comparison of the PBN results to Boolean and ODE-based modelling can be found in S6 File. Third, we demonstrated that we can study the importance of crosstalk interactions within the PBN framework by analysing the fitting quality after adding an individual interaction. Also, the weights of interaction can be derived quantitatively. Such results lead to the generation of new hypotheses on the importance of crosstalk interactions that can subsequently be tested experimentally. Lastly, we showed that we can predict the signalling profiles of combined perturbation experiments based on a PBN model which was only trained on single perturbation experiments. This may be a promising avenue for future studies to assess the effects of combinatorial perturbations or drugs treatment by performing *in silico* prediction on PBN models prior to wet-lab experiments.

### Unresolved issues and open questions from the study

There are several unresolved issues and open questions from the study. First, we observed in our initial experimental that pAKT was up-regulated after U0126 treatment (significant in DV-WT mutant). Some evidences were shown that MEK1,2 inhibition can lead to AKT up-regulation but it was only demonstrated in breast and lung cancer models [48,49]. In addition, this observation was not significant in the other two mutants in our initial set of experiment. Second, Wortmannin was shown to potentially inhibit MEK1 (MKK1) [47] and this interaction could replace the need of crosstalk interactions to fit our datasets (results not shown). However, the study is based on in-vitro kinase assays so the threshold for the inhibitory effect might differ substantially comparing to our cell-based experiments. Furthermore, other studies failed to detect this potential inhibitory effect of Wortmannin on MEK1 [46] and [50]. Third, we found that the model variant without any crosstalk interaction also has a relatively good predictive power. One might argue that we do not need to investigate the importance of crosstalk interactions nor to include them in the model. However, we still believe that crosstalk interaction is an essential element that could not be left out from the models of signal transduction networks. To support our argument, we showed in Fig 6 that at least one essential crosstalk interaction is required in the model to explain the observed signalling profiles. In addition, recent literature supports the role of crosstalk interactions in providing a compensatory mechanism within signal transduction networks which was proven to be an important factor for the discovery and design of therapeutic agents [51].

### Technical issues, limitations, and outlook on PBN modelling

There are some technical issues and limitations on the current implementation of PBN modelling that should be noted. For instance, the assignment of Boolean rules in our PBN model was still performed manually. Hence, the rules might be inconsistent between different modellers. Also, the assignment of importance on main pathways over crosstalk interactions might be biased, especially once dealing with less well-studied signalling pathways. On the technical side, *optPBN* generated results based on an approximation of the steady-state distributions. The approximation error might not be negligible for certain examples and the respective parameters (precision and number of optimisation rounds) would have to be tuned accordingly. In addition, even if the grid-based pipeline of *optPBN* can generate optimisation results in an acceptable timeframe, large computational resources are needed to perform the calculation. This might consider to be a rate-limiting step in the workflow once there is a limited amount of computational resources. This issue also posts a big challenge for analysing larger signalling networks in the future.

To account for these issues and limitations, we foresee several potential improvements to be implemented in the *optPBN* toolbox. First of all, we plan to build a standardised pipeline for converting network interactions into PBN model structures. Such pipeline should eliminate the variability of PBN modelling among different modellers. In terms of computation, a recent PBN simulation tool on Java called “ASSA-PBN” was developed which allows for a more precise steady-state approximation and for faster calculations [52]. These features of ASSA-PBN could be integrated into the *optPBN* framework in order to improve the efficiency and accuracy of the results. On another perspective, PBN models might also be compiled into executable scripts to gain a significant computational speed-up, similar to ODE model compilation provided by the Systems Biology toolbox 2 (SBTB2) [45].

As for an outlook into future applications, we envisage that the same analytical pipeline as presented here could also be applied to study the properties of different signalling networks at steady-state. Furthermore, since PBN is also widely used to model gene regulatory networks, it is possible to build multi-scale models which connect the two functional layers within the PBN framework in order to get more comprehensive insights.

## Conclusion

We demonstrated a novel pipeline for modelling and analysing signal transduction networks with steady-state data applying the PBN approach. By integrating normalised measurement data at steady-state into an *a priori* model topology derived from literature using the tool *optPBN*, we could generate a PBN model which fits well to signalling data at steady-state quantitatively. An additional experiment was performed to investigate the validity of predicted crosstalk interactions. After a new round of experimental and modelling work, we generated a final PBN model of the PDGF signalling network contextualized for GIST with a high predictive power for predicting the combinatorial treatments from the individual stimulation measurements. Such model could be further used to guide and reduce the number of experimental conditions to be investigated in the lab. In addition, the analytical pipeline we presented could also be used to study and analyse other signal transduction networks [53].

## Supporting Information

S1 Fig Expression level of signalling molecules in PDGFRα mutants. A panel of PDGFRα-D842V mutants with YF point mutations in a separated set of experiment is shown. The experiments were performed following the same protocol as described in Materials and Methods. We observed that different constructions of mutants, including the Y720F and YY731/742FF mutants in our study, did not affect the expression level of the signalling molecules that we investigated.
(PDF)

S2 Fig Compared fitting quality of the initial PBN variants with all-or-none inhibition versus with partial inhibition. Model simulations from two initial PBN model variants, one with all-or-none inhibition and another with partial inhibition, were compared against the training dataset in 6 experimental conditions. The model variant with all-or-none inhibition could not be fitted to the data points of pPLCγ and pERK1,2 in DV-dMAPK condition while the model variant with partial inhibition fitted well to these data points. Nevertheless, the latter model still could not distinguish the differences in pERK1,2 signals comparing between DV-WT and DV-WT-Wortmannin conditions.
(PDF)

S3 Fig Predictive power of the final PBN model versus the model variant without crosstalk. Model simulations from the final model and the refined model variant without any crosstalk interaction were compared against the validation dataset in 4 experimental conditions. Both model variants have very good predictive power on most of the experimental conditions. Nevertheless, the refined model variant without any crosstalk interaction could not accurately predict the change of pERK1,2 signal in DV-dPI3K-Wort condition which is modulated via crosstalk signalling.
(PDF)

S4 Fig Effect of single perturbation experiment removal on model prediction. The distributions of output state values from 20 optimisation runs of the final model and the model variants with a single perturbation experiment removed one-at-a-time were compared against the validation dataset in 4 experimental conditions. The model variants with single perturbation experiment removed have broader ranges of predicted output values (highlighted with red boxes) where some do not agree well with the experimental data in the validation dataset.
(PDF)

S1 File Model description of the PDGF signalling network in PBN format. This document provides a detailed description of the PBN models of PDGF signalling presented in our study. The model descriptions are divided into 3 sections: 1) The core model structure with major intracellular signalling pathways, 2) The integration of crosstalk interactions proposed in literature, and 3) The final model structure after integrating data from the additional experiment. Small examples within the computational scripts are also included in each section to illustrate how each type of network interaction can be coded in the PBN format.
(PDF)

S2 File Computational scripts of the PBN models and the result files from the modelling studies. This compressed zip file comprises the following elements from modelling study: 1) PBN model descriptions in the form of computational scripts, 2) Examples of result file from grid-based computation, and 3) Saved model structures for grid computation. Instructions on how to further analyse the result files and how to re-perform the optimisation are included.
(ZIP)

S3 File Model complexities, F-test and fitting cost of model variants from 3 optimisation runs. This spreadsheet provides the number of data points and the number of optimised parameters for each model variant. In addition, the fitting costs from 3 optimisation runs of each model variants are provided where only small variations between runs were observed. The calculated AIC values and F values from F-test for regression model are also shown.
(XLSX)

S4 File Fitting costs from pair-wise crosstalk combination study. This spreadsheet provides the fitting costs of the model variants with pairs of combined crosstalk interactions comparing to the ones with single crosstalk interaction (in diagonal) and without crosstalk interaction. Only the model variants that include crosstalk number 3 (from PI3K to Ras) or number 4 (from PI3K to MEK1,2) have better fitting cost.
(XLSX)

S5 File Weights of interaction of the final PBN model. This spreadsheet provides a list of Boolean interactions in the final PBN model together with the mean and standard deviation of the optimised selection probabilities, i.e. weights of interaction, generated from the best 500 parameter sets of the optimisation. Very low overall standard deviations of the optimised weights (mean = 0.03) indicate the high consistency of the optimisation result.
(XLS)

S6 File Comparison of PBN versus Boolean network and ODE-based modelling results. This document presents a comparison of PBN versus two other modelling frameworks which are Boolean network and ODE-based modelling. This additional study includes model fitting and predictions as well as a comparison of results from the three modelling approaches.
(PDF)
