# Supplementary figures and images for: A Probabilistic Boolean Network Approach for the Analysis of Cancer-Specific Signalling: A Case Study of Deregulated PDGF Signalling in GIST
Source: PLoS One. 2016 May 27;11(5):e0156223. doi: 10.1371/journal.pone.0156223 (PMC4883749; doi:10.1371/journal.pone.0156223)

# PDGFR $\alpha$ D842V

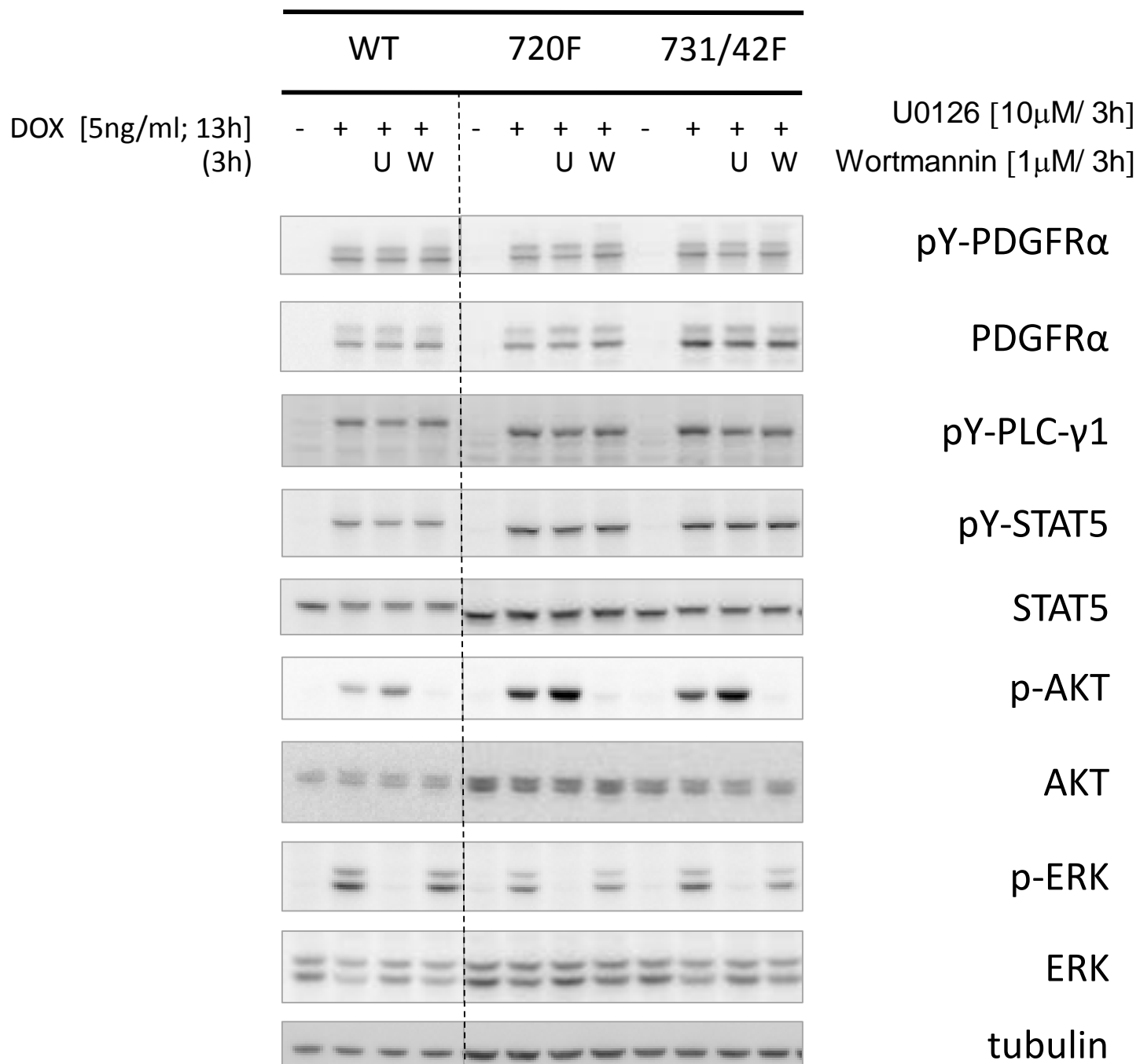

The represented blots were cut for representation purpose (dashed line)

Supplement: S1 Fig — A panel of PDGFRα-D842V mutants with YF point mutations in a separated set of experiment is shown. The experiments were performed following the same protocol as described in Materials and Methods. We observed that different constructions of mutants, including the Y720F and YY731/742FF mutants in our study, did not affect the expression level of the signalling molecules that we investigated. (PDF) [file pone.0156223.s001.pdf]
