# Supplementary material for: A Probabilistic Boolean Network Approach for the Analysis of Cancer-Specific Signalling: A Case Study of Deregulated PDGF Signalling in GIST
Source: PLoS One. 2016 May 27;11(5):e0156223. doi: 10.1371/journal.pone.0156223 (PMC4883749; doi:10.1371/journal.pone.0156223)
Supplement: S2 Fig — Model simulations from two initial PBN model variants, one with all-or-none inhibition and another with partial inhibition, were compared against the training dataset in 6 experimental conditions. The model variant with all-or-none inhibition could not be fitted to the data points of pPLCγ and pERK1,2 in DV-dMAPK condition while the model variant with partial inhibition fitted well to these data points. Nevertheless, the latter model still could not distinguish the differences in pERK1,2 signals comparing between DV-WT and DV-WT-Wortmannin conditions. (PDF) [file pone.0156223.s002.pdf]

All-or-none inhibition (no crosstalk) / Fitting cost: 0.802

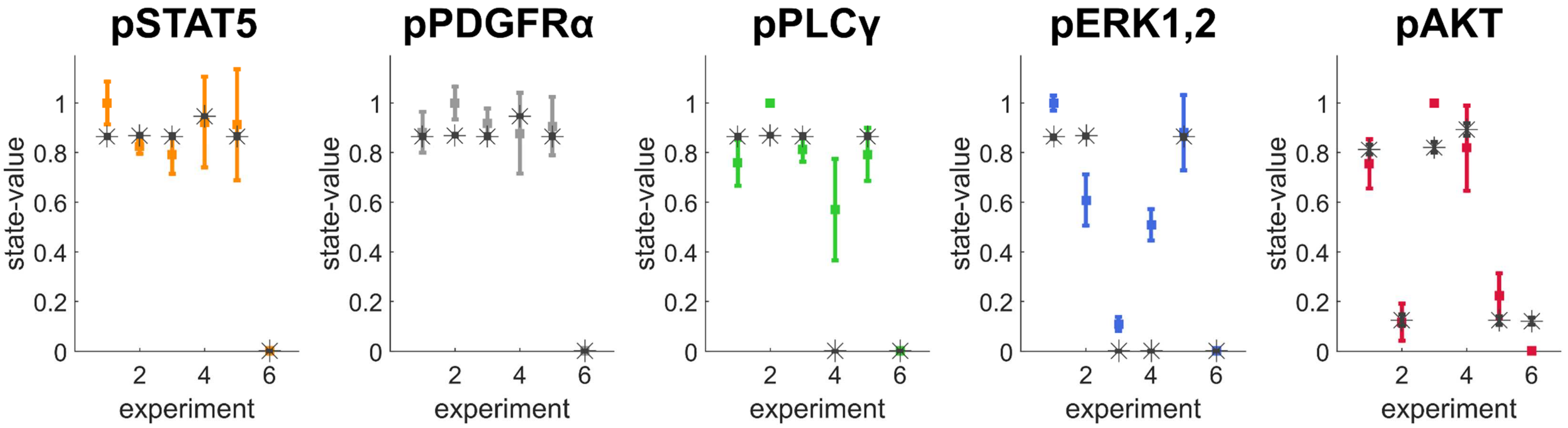

Partial inhibition (no crosstalk) / Fitting cost: 0.198

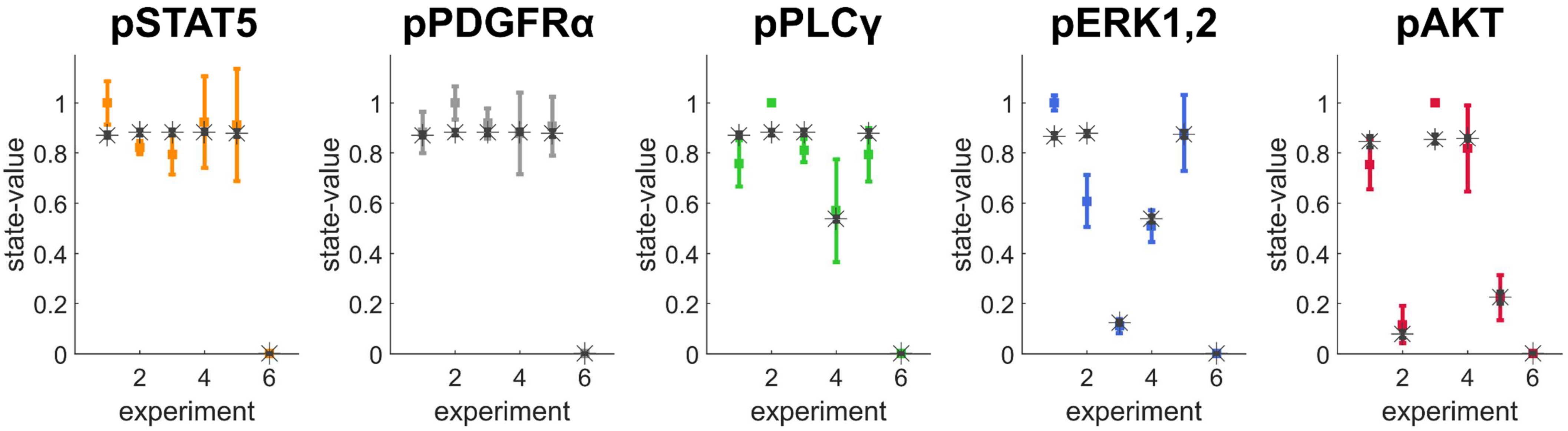

Annotation on x-axis: 1 = DV-WT, 2 = DV-WT-Wort., 3 = DV-WT-U0126, 4 = DV-dMAPK, 5 = DV-dPI3K, 6 = neg ct.
