# Supplementary material for: A Probabilistic Boolean Network Approach for the Analysis of Cancer-Specific Signalling: A Case Study of Deregulated PDGF Signalling in GIST
Source: PLoS One. 2016 May 27;11(5):e0156223. doi: 10.1371/journal.pone.0156223 (PMC4883749; doi:10.1371/journal.pone.0156223)
Supplement: S3 Fig — Model simulations from the final model and the refined model variant without any crosstalk interaction were compared against the validation dataset in 4 experimental conditions. Both model variants have very good predictive power on most of the experimental conditions. Nevertheless, the refined model variant without any crosstalk interaction could not accurately predict the change of pERK1,2 signal in DV-dPI3K-Wort condition which is modulated via crosstalk signalling. (PDF) [file pone.0156223.s003.pdf]

Model variant without crosstalk interaction

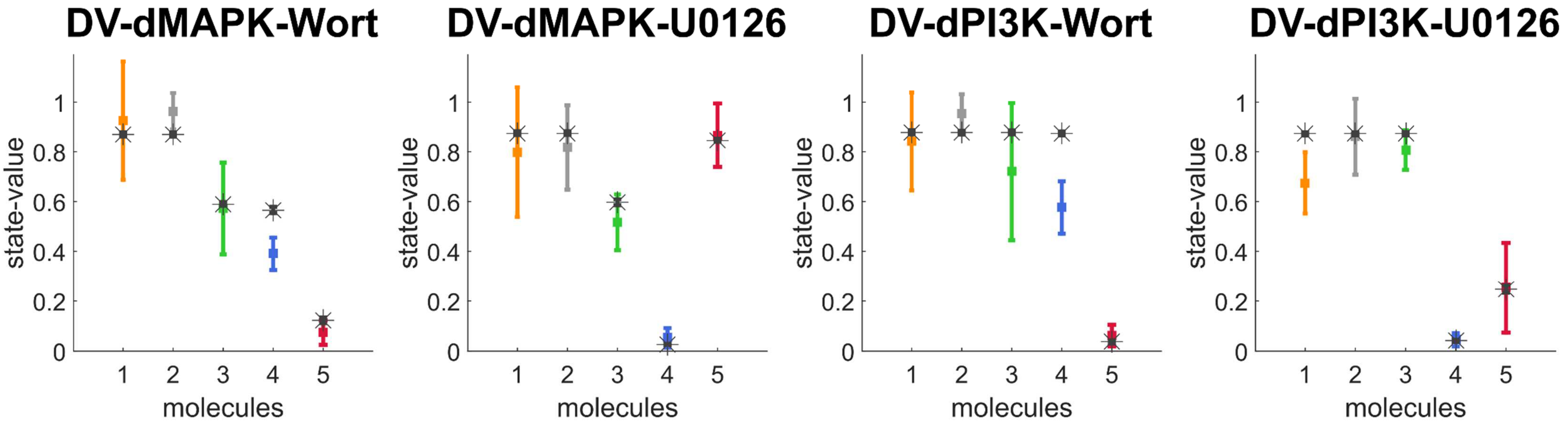

Final model (with PI3K->MEK1,2 interaction)

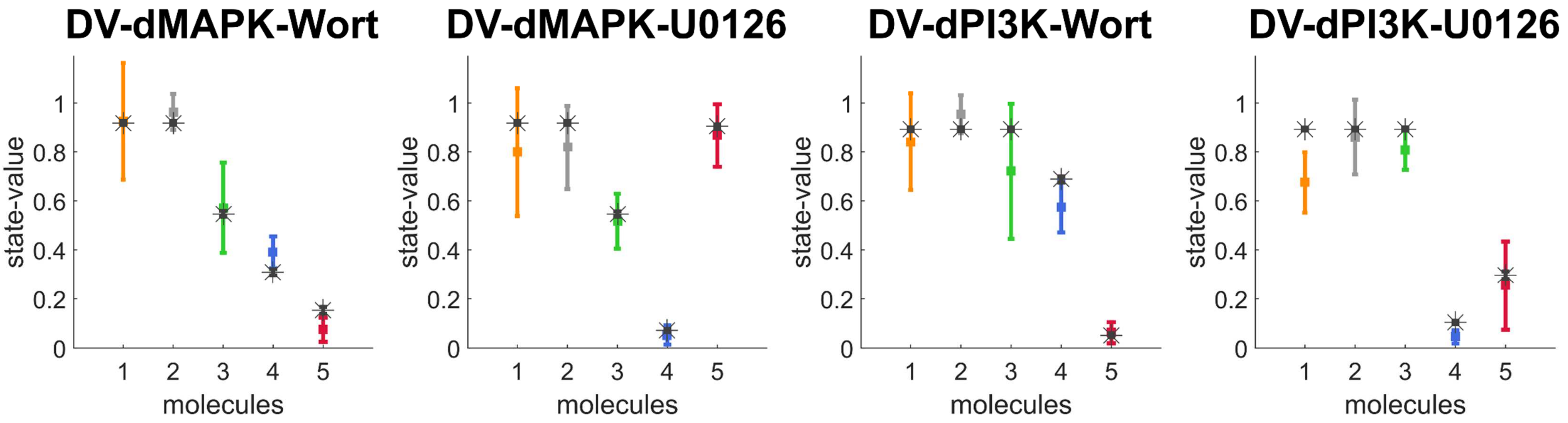

Annotation on x-axis: 1 = pSTAT5, 2 = pPDGFRα, 3 = pPLCγ, 4 = pERK1,2, 5 = pAKT
