# Supplementary material for: A Probabilistic Boolean Network Approach for the Analysis of Cancer-Specific Signalling: A Case Study of Deregulated PDGF Signalling in GIST
Source: PLoS One. 2016 May 27;11(5):e0156223. doi: 10.1371/journal.pone.0156223 (PMC4883749; doi:10.1371/journal.pone.0156223)
Supplement: S4 Fig — The distributions of output state values from 20 optimisation runs of the final model and the model variants with a single perturbation experiment removed one-at-a-time were compared against the validation dataset in 4 experimental conditions. The model variants with single perturbation experiment removed have broader ranges of predicted output values (highlighted with red boxes) where some do not agree well with the experimental data in the validation dataset. (PDF) [file pone.0156223.s004.pdf]

Effect of single perturbation experiment removal on model prediction

All single perturbation

No Exp2 (No Wort)

No Exp3 (No U0126)

No Exp4 (No dMAPK)

No Exp7 (No dPI3K)

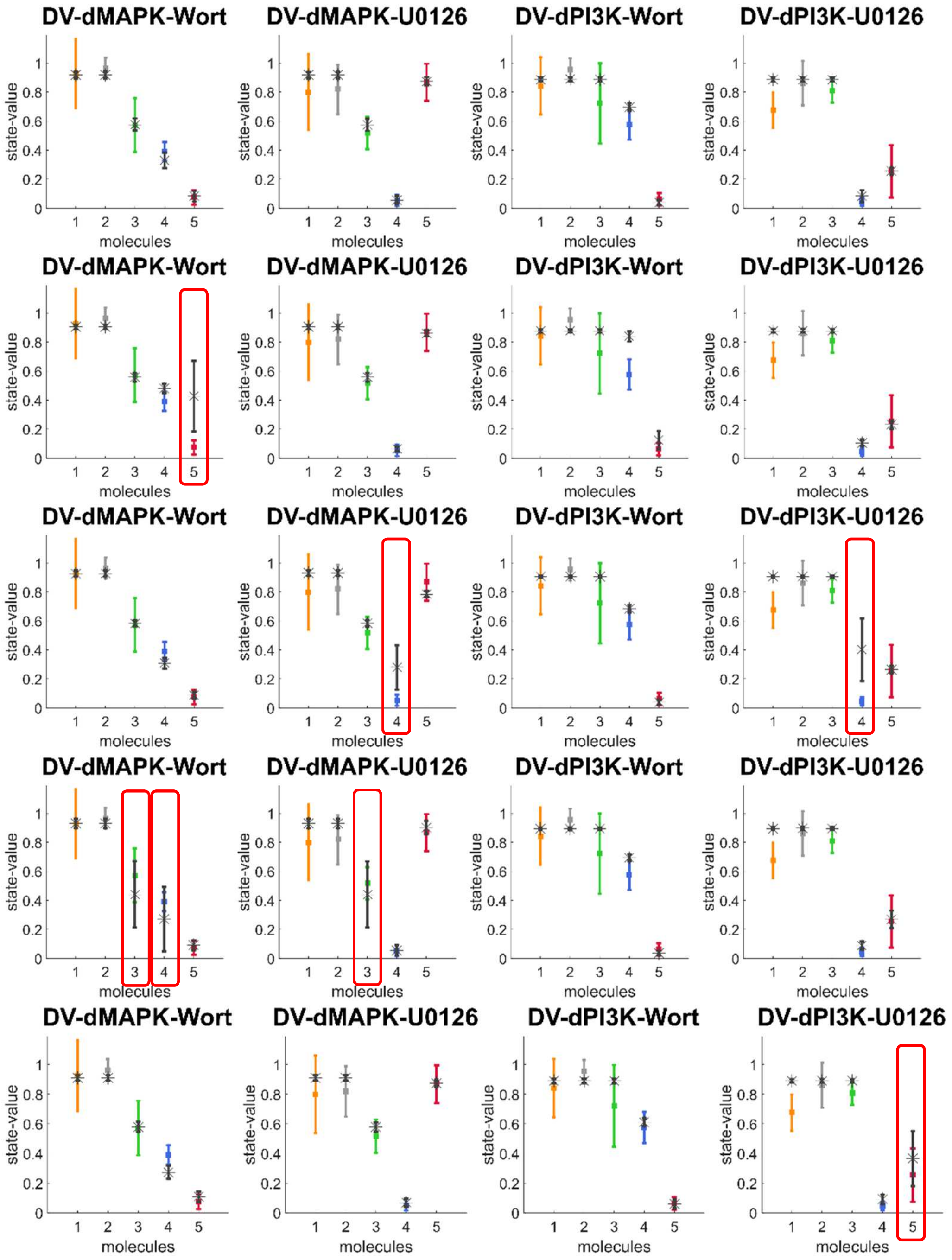

Annotation on x-axis: 1 = pSTAT5, 2 = pPDGFRα, 3 = pPLCγ, 4 = pERK1,2, 5 = pAKT
