# Supplementary material for: A Probabilistic Boolean Network Approach for the Analysis of Cancer-Specific Signalling: A Case Study of Deregulated PDGF Signalling in GIST
Source: PLoS One. 2016 May 27;11(5):e0156223. doi: 10.1371/journal.pone.0156223 (PMC4883749; doi:10.1371/journal.pone.0156223)
Supplement: S1 File — This document provides a detailed description of the PBN models of PDGF signalling presented in our study. The model descriptions are divided into 3 sections: 1) The core model structure with major intracellular signalling pathways, 2) The integration of crosstalk interactions proposed in literature, and 3) The final model structure after integrating data from the additional experiment. Small examples within the computational scripts are also included in each section to illustrate how each type of network interaction can be coded in the PBN format. (PDF) [file pone.0156223.s005.pdf]

Supporting Information File S1:

Model description of the PDGF signalling network in PBN format

---

This document provides a detailed description of the PBN models of PDGF signalling which are presented in the manuscript. The model descriptions are separated into 3 parts as follows:

1) The core model structure with major intracellular signalling pathways
2) The integration of crosstalk interactions which were proposed in literature
3) The refined model structure after integrating data from additional experiment

Please note that we applied the annotation '->' to depict an activating interaction and '-|' to depict an inhibitory interaction.

Part 1: The core model structure with major intracellular signalling pathways

We built a core model structure of PDGF signalling comprising PDGFRα and its regulatory mechanisms together with the major downstream signalling pathways including MAPK, PI3K/AKT/mTOR, PLCγ/PKC pathways as well as STAT5. The core model topology is shown below:

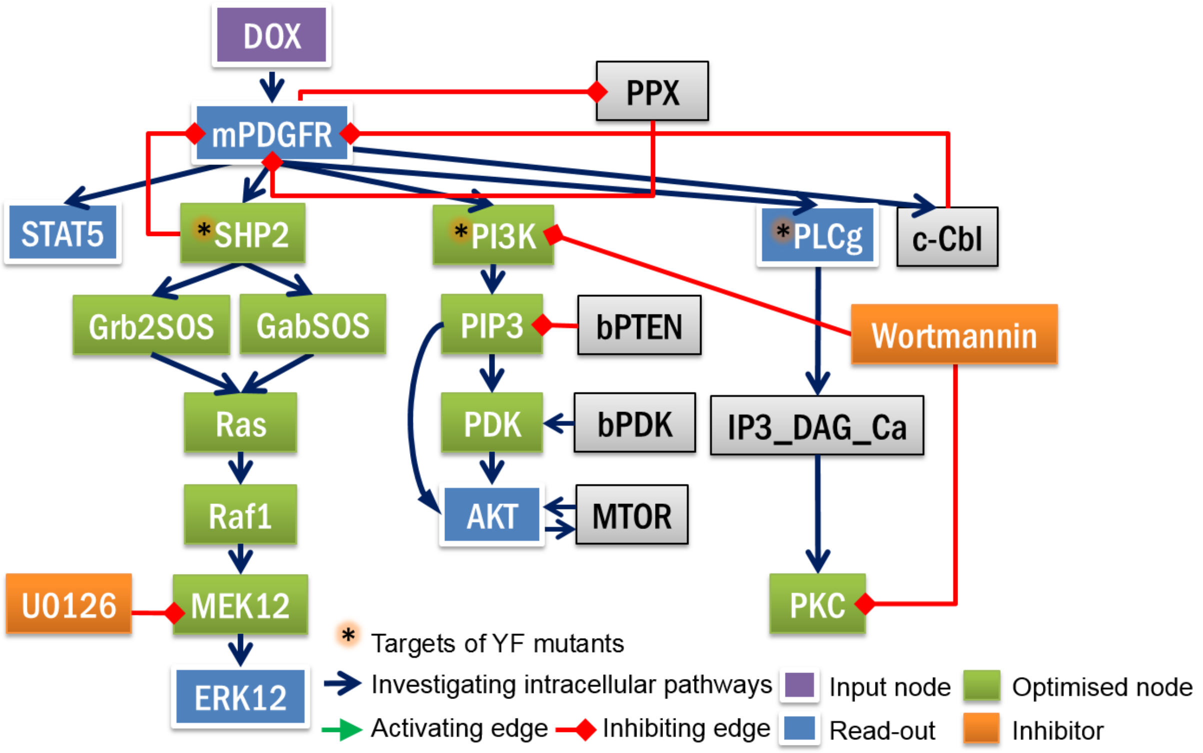

From the top, we depicted the induction of PDGFRα-transgene by doxycycline (DOX) in our cell system. The regulatory mechanisms of mutated PDGFRα (mPDGFR, represented as 'PDGFR' in the

model) are governed by cytoplasmic phosphatase (PPX), protein tyrosine phosphatase shp2 (SHP2) and proteosomal degradation via ubiquitination promoted by c-Cbl (cCbl). The network topology of downstream signalling pathways comprises as follows:

- MAPK pathway (SHP2 -> Grb2SOS/GabSOS -> Ras -> Raf1 -> MEK1,2 (MEK12) -> ERK1,2 (ERK12))
- PI3K-AKT-mTOR pathway (PI3K -> PIP3 -> PDK -> AKT <-> mTOR) with basal activity of PTEN (bPTEN) inhibiting PIP3 and basal activity of PDK (bPDK) activating PDK. Note that PIP3 can also directly activate Akt.
- PLCg-PKC pathway (PLCg -> IP3_DAG_Ca -> PKC)
- STAT5

According to this model topology, we formulated the model descriptions with Boolean rules as follows:

```
=== PDGFR activation and regulatory mechanisms ===
% DOX:              rules=[rules; {'DOX = 1','1','C'}];
% PPX:              rules=[rules; {'PPX = ~PDGFR','1','C'}];
% cCbl:             rules=[rules; {'cCbl = PDGFR','1','C'}];
% PDGFR:            rules=[rules; {'PDGFR = DOX','1','D'}];
                    rules=[rules; {'PDGFR = DOX & ~cCbl','1','D'}];
                    rules=[rules; {'PDGFR = DOX & ~PPX','1','D'}];
                    rules=[rules; {'PDGFR = DOX & ~SHP2','1','D'}];

=== MAPK pathway ===
% SHP2:             rules=[rules; {'SHP2 = PDGFR','1','C'}];
% Grb2SOS:          rules=[rules; {'Grb2SOS = SHP2','1','C'}];
% GabSOS:           rules=[rules; {'GabSOS = SHP2','1','C'}];
% Ras:              rules=[rules; {'Ras = Grb2SOS | GabSOS','1','C'}];
% Raf1:             rules=[rules; {'Raf1 = Ras','1','C'}];
% MEK12:            rules=[rules; {'MEK12 = MEK12_induce','1','C'}];
% MEK12_induce:     rules=[rules; {'MEK12_induce = Raf1','1','C'}];
% ERK12:            rules=[rules; {'ERK12 = MEK12','1','C'}];

=== PI3K-AKT-mTOR pathway ===
% PI3K_PDGFR:       rules=[rules; {'PI3K_PDGFR = PDGFR','1','C'}];
% prePI3K:          rules=[rules; {'prePI3K = PI3K_PDGFR','1','C'}];
% PI3K:             rules=[rules; {'PI3K = prePI3K','1','C'}];
% bPTEN:            rules=[rules; {'bPTEN = 1','1','C'}];
% PIP3:             rules=[rules; {'PIP3 = PI3K','1','D'}];
                    rules=[rules; {'PIP3 = PI3K & ~bPTEN','1','D'}];
% bPDK:             rules=[rules; {'bPDK = 1','1','C'}];
% PDK:              rules=[rules; {'PDK = PIP3','1','D'}];
                    rules=[rules; {'PDK = bPDK','1','D'}];
% Akt:              rules=[rules; {'Akt = PIP3','1','D'}];
                    rules=[rules; {'Akt = PDK','1','D'}];
                    rules=[rules; {'Akt = MTOR','1','D'}];
% MTOR:             rules=[rules; {'MTOR = Akt','1','C'}];

=== PLCg-PKC pathway ===
% PLCg:             rules=[rules; {'PLCg = PDGFR','1','C'}];
% IP3_CaIon_DAG:    rules=[rules; {'IP3_CaIon_DAG = PLCg','1','C'}];
```

```
% PKC_induce:        rules=[rules; {'PKC_induce = IP3_CaIon_DAG','1','C'}];
% PKC:               rules=[rules; {'PKC = PKC_induce','1','C'}];

  === STAT5 ===
% STAT5:             rules=[rules; {'STAT5 = PDGFR','1','C'}];
```

Based on these assigned Boolean rules, there are a few important observations. First, the flags ‘C’ and ‘D’ at the end of the Boolean rules are defined to correlate with the terms ‘Constant’ and ‘Default’ respectively. Once the flag ‘C’ is used, it means that the selection probability of that Boolean rule will be constant and the corresponding node (molecule) will not be optimised so all rules for this node need to have the flag ‘C’ then. This flag is exclusively applied for the nodes with only one input as the probability of activating or inhibiting such nodes have to be 1. In parallel, when a node has more than one incoming interaction, multiple Boolean rules can be assigned for such node and the respective node is typically optimised. The flag ‘D’ can be assigned throughout multiple Boolean rules on a single node to give a full boundary of the selection probability from 0 to 1 during the optimisation.[1] With this flag assigned, each regulatory mechanism as coded in different Boolean rule will have an equal chance to dominate the other. In general, this assignment is defined once the influence from each interaction is unclear. For instance, the activation of Akt can be mediated by PIP3, PDK and mTOR but there is no prior information nor assumption whether one specific source of signal will be stronger than the others.

Another observation that should be noted is some molecules, i.e. MEK1,2, PI3K and PKC have intermediate nodes. These nodes represent the intermediate steps of signal transduction processes where multiple activating and inhibiting interactions take place. On the last observation on the inhibitory mechanisms, we applied 2 types of single perturbation to the system, i.e. additional YF point mutations to abrogate recruitment sites on the PDGFRα (dMAPK and dPI3K) and signalling inhibitors (Wortmannin and U0126) in the initial experiment. Based on our experimental observations and literature information, we found that the abrogation of tyrosine residue Y720F in dMAPK mutant also affects the binding of pPLCg to PDGFRα while Wortmannin has an additional off-target on PKC apart from inhibiting PI3K. To account for these inhibitory mechanisms without adding more nodes, we repeated the same Boolean rule for the activating interaction once and we set the second Boolean rule as 0 once the targeted molecule is inhibited. To illustrate our methodology, we provide an example of Boolean rules for the D842V-PDGFRα mutant without YF point mutation or signalling inhibitor (DV-WT) compared to the Boolean rules of D842V-PDGFRα-dMAPK mutant and Wortmannin treatment (DV-dMAPK-Wort.) as follows:

```
=== DV-WT ===
% SHP2:              rules=[rules; {'SHP2 = PDGFR','1','D'}];
                     rules=[rules; {'SHP2 = PDGFR','1','D'}];
% MEK12:             rules=[rules; {'MEK12 = MEK12_induce','1','D'}];
                     rules=[rules; {'MEK12 = MEK12_induce','1','D'}];
% PI3K_PDGFR:        rules=[rules; {'PI3K_PDGFR = PDGFR','1','D'}];
                     rules=[rules; {'PI3K_PDGFR = PDGFR','1','D'}];
% prePI3K:           rules=[rules; {'prePI3K = PI3K_PDGFR','1','C'}];
```

[1] If the sum of optimised selection probabilities from multiple Boolean rules is larger than 1, the selection probability of each Boolean rule will be normalised to the sum of all probabilities in order to ensure that the sum of the selection probabilities will be 1.

```
% PI3K:          rules=[rules; {'PI3K = prePI3K','1','D'}];
                 rules=[rules; {'PI3K = prePI3K','1','D'}];
% PLCg:          rules=[rules; {'PLCg = PDGFR','1','D'}];
                 rules=[rules; {'PLCg = PDGFR','1','D'}];
% PKC:           rules=[rules; {'PKC = PKC_induce','1','D'}];
                 rules=[rules; {'PKC = PKC_induce','1','D'}];
```

=== DV-dMAPK-Wort. ===

```
% SHP2:          rules=[rules; {'SHP2 = PDGFR','1','D'}];
                 rules=[rules; {'SHP2 = 0','1','D'}]; % effect of dMAPK
% MEK12:         rules=[rules; {'MEK12 = MEK12_induce','1','D'}];
                 rules=[rules; {'MEK12 = MEK12_induce','1','D'}];
% PI3K_PDGFR:    rules=[rules; {'PI3K_PDGFR = PDGFR','1','D'}];
                 rules=[rules; {'PI3K_PDGFR = PDGFR','1','D'}];
% prePI3K:       rules=[rules; {'prePI3K = PI3K_PDGFR','1','C'}];
% PI3K:          rules=[rules; {'PI3K = prePI3K','1','D'}];
                 rules=[rules; {'PI3K = 0','1','D'}]; % effect of Wortmannin
% PLCg:          rules=[rules; {'PLCg = PDGFR','1','D'}];
                 rules=[rules; {'PLCg = 0','1','D'}]; % effect of dMAPK
% PKC:           rules=[rules; {'PKC = PKC_induce','1','D'}];
                 rules=[rules; {'PKC = 0','1','D'}]; % effect of Wortmannin
```

As demonstrated, the second rules of SHP2 and PLCg were changed to 0 to account for the inhibitory effect of dMAPK while the second rules of PI3K and PKC were also set to 0 to take the inhibitory effect of Wortmannin into consideration. A similar assignment was applied to the second rule of PI3K_PDGFR in order to account for the dPI3K effect, and it was also applied to the second rule of MEK12 in order to account for U0126 inhibition. With the methodology for the assignment of Boolean rules as demonstrated, we were able to generate the model structures of PDGF signalling which could represent the six experimental conditions within the training dataset and we subsequently applied them to perform model fitting accordingly.

Part 2: The integration of crosstalk interactions which were proposed in literature

After we tried to fit the core model topology to the training dataset, we discovered that the model fitted to the data relatively well but there were still a few data points where could not be fitted. We hypothesise that a certain number of crosstalk interactions is required to explain all data points. Therefore, we built 9 additional model variants by adding the crosstalk interactions which were proposed in the literature to the core model topology one-at-a-time and we re-performed the optimisation on the model variants to the training dataset. The types and targets of the crosstalk interactions in the 9 model variants are shown as follows:

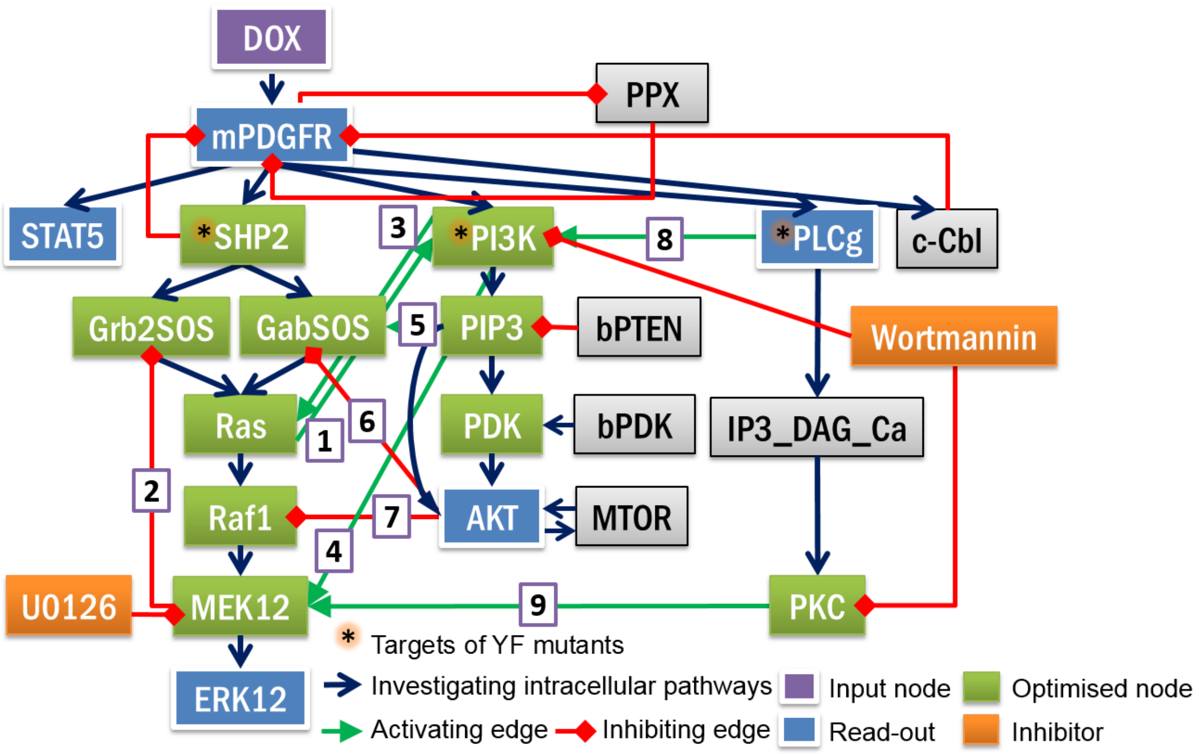

In terms of the implementation in the PBN model, one interaction was added to the core model topology (Part 1) for each model variant. For instance, the model variant with crosstalk number 9 (PKC -> MEK12) has the following Boolean rules:

```
% MEK12_induce:    rules=[rules; {'MEK12_induce = Raf1','1','H'}];
                   rules=[rules; {'MEK12_induce = PKC','1','L'}];
```

Note that another set of flags ‘H’ and ‘L’ were used. As previous mentioned in the manuscript, we consider that the signals flow through canonical pathways stronger than flowing pass crosstalk interactions. Hence, we assign the flag ‘H’ (High) for the canonical interaction Raf1 -> MEK12 and the flag ‘L’ (Low) for the crosstalk interaction PKC -> MEK12. This assignment ensure that the boundary of the interaction(s) with the flag ‘H’ will always have higher selection probability compared to the interaction(s) with the flag ‘L’.

Among the pool of all model variants, one of the complex model variants that should be mentioned is the model variant with crosstalk number 1 (Ras -> PI3K). In this model variant, we introduce a new node called ‘PI3K_crosstalk’ coded as “rules=[rules; {'PI3K_crosstalk = Ras','1','C'}];” to account for this crosstalk interaction. Previously, we had the node ‘PI3K_PDGFR’ which represents PI3K signal after the activation by PDGFR. To integrate the signals, the successive node ‘prePI3K’ was assigned to represent PI3K signals which receive the inputs both from PDGFR and from crosstalk interaction(s) as depicted in following Boolean rules:

```
% prePI3K:         rules=[rules; {'prePI3K = PI3K_PDGFR','1','H'}];
                   rules=[rules; {'prePI3K = PI3K_crosstalk','1','L'}];
```

Note that the information on canonical pathway versus crosstalk interaction is also applied here with the flags ‘H’ and ‘L’. Subsequently, the integrated signals of PI3K in ‘prePI3K’ node can further be inhibited by Wortmannin by setting the second rule of ‘PI3K’ node to 0 as explained above.

Part 3: The refined model structure after integrating data from the additional experiment

After we performed the additional experiment (Figure 3) and we found that PKC activity is independent of the activation from PDGFRα, we added the node 'bPKC' to account for the basal activity of PKC with the following Boolean rules:

```
% PKC_induce:        rules=[rules; {'PKC_induce = IP3_CaIon_DAG','1','D'}];
                     rules=[rules; {'PKC_induce = bPKC','1','D'}];
```

The updated model topology with the additional information on two new PKC inhibitors (GF109 and Gö6976) is shown in the figure below:

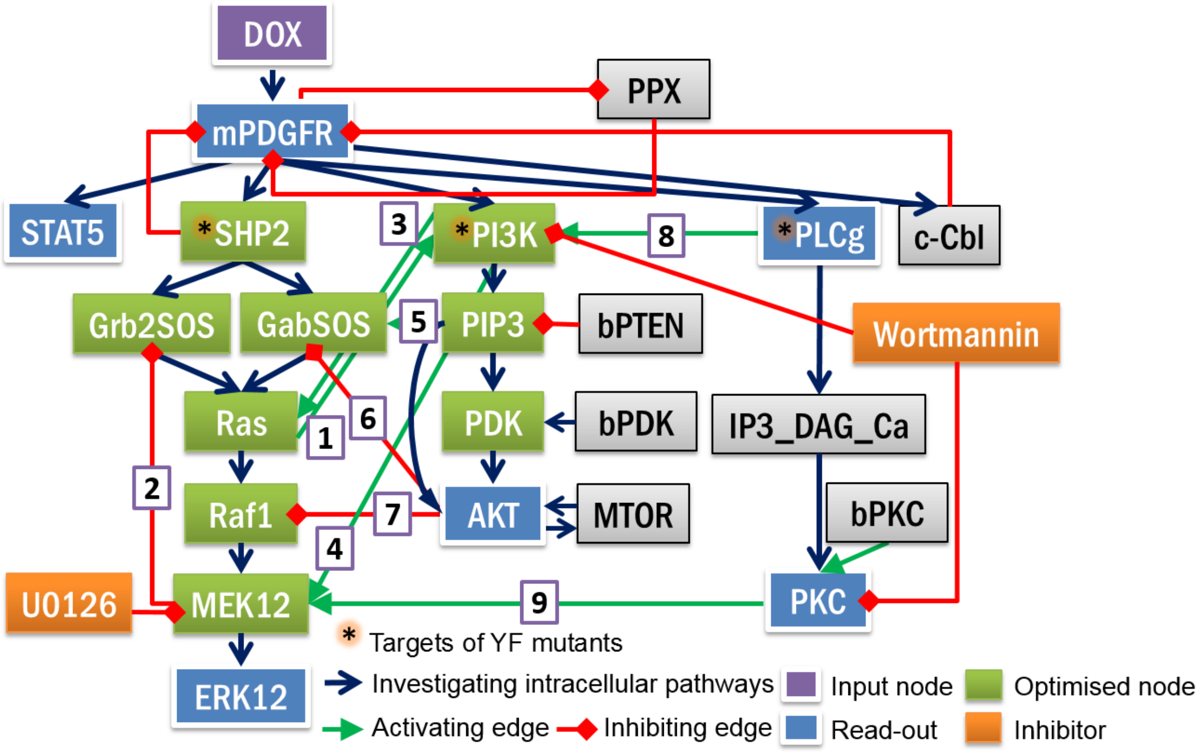

Note that even if we performed an additional experiment with the two new PKC inhibitors, we only took the information from the experimental conditions which overlapped with the ones in the training dataset for modelling. With this regard, we only included pPKC substrates data on negative control, positive control (DV-WT) and DV-WT-Wortmannin conditions into the training dataset for final model refinement.

After we fitted the refined model to the updated training dataset, we also performed an *in silico* analysis to investigate the importance of crosstalk interactions which were described in Step 2. This analysis revealed that only crosstalk interactions number 3 (PI3K -> Ras) or number 4 (PI3K -> MEK1,2) are important to fit the training dataset. Then, we proceeded with the final PBN model, i.e. the refined model plus the crosstalk interaction number 4 (PI3K -> MEK1,2) to evaluate the predictive power of the model. We found that the final model could predict the signalling profiles in the combined perturbation conditions within the validation dataset accurately. Hence, we concluded that our final PBN model is highly predictive.
