# Supplementary material for: A Probabilistic Boolean Network Approach for the Analysis of Cancer-Specific Signalling: A Case Study of Deregulated PDGF Signalling in GIST
Source: PLoS One. 2016 May 27;11(5):e0156223. doi: 10.1371/journal.pone.0156223 (PMC4883749; doi:10.1371/journal.pone.0156223)
Supplement: S2 File — This compressed zip file comprises the following elements from modelling study: 1) PBN model descriptions in the form of computational scripts, 2) Examples of result file from grid-based computation, and 3) Saved model structures for grid computation. Instructions on how to further analyse the result files and how to re-perform the optimisation are included. (ZIP) [file pone.0156223.s006.zip › General_Information.docx]

**Supporting Information File S2:**

Computational scripts of the PBN models and the result files from the modelling studies.

This compressed zip file consists of the following elements from the 3 phases of the modelling study:

1. PBN model descriptions (computational scripts)
2. Results from grid computation (log files)
3. Saved model structures for grid computation

For each phase of the modelling study, users can explore the evolution of optimisation results from the grid-based computation on the files with the prefix “TimeOutput”. The best fitting cost from differential evolution (DE) algorithm for each model variant was selected as representative result.

In case users would like to perform further analysis based on the optimisation results (e.g., to explore the distribution of optimised selection probabilities as shown in the optPBN study [1]), user can explore the provided examples of result file from DE algorithm for each model variant. The complete set of result files can be downloaded from the optPBN project on SourceForge [2].

If users would like to re-perform the optimisation, we invite users to download the latest grid-based version of the optPBN toolbox (v2.2.3) [2] and install it on a cluster or a grid-based infrastructure, e.g. Grid’5000 [3] (see the manual of installation in the optPBN study [1]). After installation, users can directly use the provided saved model structures for grid computation and run the optimisation on parallel processing units. The provided optimisation results for this study were generated from “Reims” site on Grid’5000 using 160 parallel processing units.

*Reference:*

1. Trairatphisan P, Mizera A, Pang J, Tantar AA, Sauter T. optPBN: an optimisation toolbox for Probabilistic Boolean Network. PLoS One. 2014;9(8): e105913. doi: 10.1371/journal.pone.0098001

2. <http://www.sourceforge.com/projects/optpbn>

3. <https://www.grid5000.fr/mediawiki/index.php/Grid5000:Home>
