# Supplementary material for: A Probabilistic Boolean Network Approach for the Analysis of Cancer-Specific Signalling: A Case Study of Deregulated PDGF Signalling in GIST
Source: PLoS One. 2016 May 27;11(5):e0156223. doi: 10.1371/journal.pone.0156223 (PMC4883749; doi:10.1371/journal.pone.0156223)
Supplement: S6 File — This document presents a comparison of PBN versus two other modelling frameworks which are Boolean network and ODE-based modelling. This additional study includes model fitting and predictions as well as a comparison of results from the three modelling approaches. (PDF) [file pone.0156223.s010.pdf]

Supporting Information S6 File:

Comparison of PBN versus Boolean network and ODE-based modelling results

We compared model complexity and optimisation results of the deregulated PDGF signalling in GIST in the PBN framework to the ones generated by Boolean networks and ODE-based models, see Table 1. The plotted output states of selected molecules are demonstrated in Fig 1.

Table 1. Comparison of model complexities and optimisation results from 3 different modelling approaches.

	BN (BN/PBN)	PBN (optPBN)	ODE (SBTB2)
Number of nodes	23	27	24
Number of rules/reactions	23	41 to 42	45
Number of optimised parameters	None	25 to 27	51 to 53
Best optimal cost (no CT)	1.206	0.208	0.324
AIC value (no CT)	N/A	-117.20	-50.56
Best optimal cost (with CT4)	1.188	0.162	0.253
AIC value (with CT4)	N/A	-121.45	-54.77

The model complexities (sizes, interactions, and optimised parameters) as well as the fitting costs and the AIC values of two different model variants, i.e. a revised model without crosstalk (no CT) and the final model with the essential crosstalk number 4 (CT4) from PI3K to MEK1,2, generated by 3 modelling approaches are compared. The computational tools used for the analysis are shown in parentheses next to the names of modelling frameworks. The ranges of rules/reactions and of optimised parameters, if exist, indicate the numbers for the model variant without crosstalk and with the essential crosstalk number 4, respectively.

It was shown that Boolean modelling yields the smallest network as only one Boolean rule is assigned for each node. Because of its simplicity, different types of interactions are combined and influence the target node in an all-or-none fashion, resulting in state values of 0 or 1. Such discrete state values do not fit well to the normalised experimental data in our setting and lead to a high fitting cost. Even if the experimental data were discretised to 0 or 1 using the cut-off of 0.5 prior to the comparison, the model in the Boolean network framework still could not correctly describe the partial signal abrogation of pPLCγ in the DV-dMAPK mutant and return a high optimal cost (data not shown).

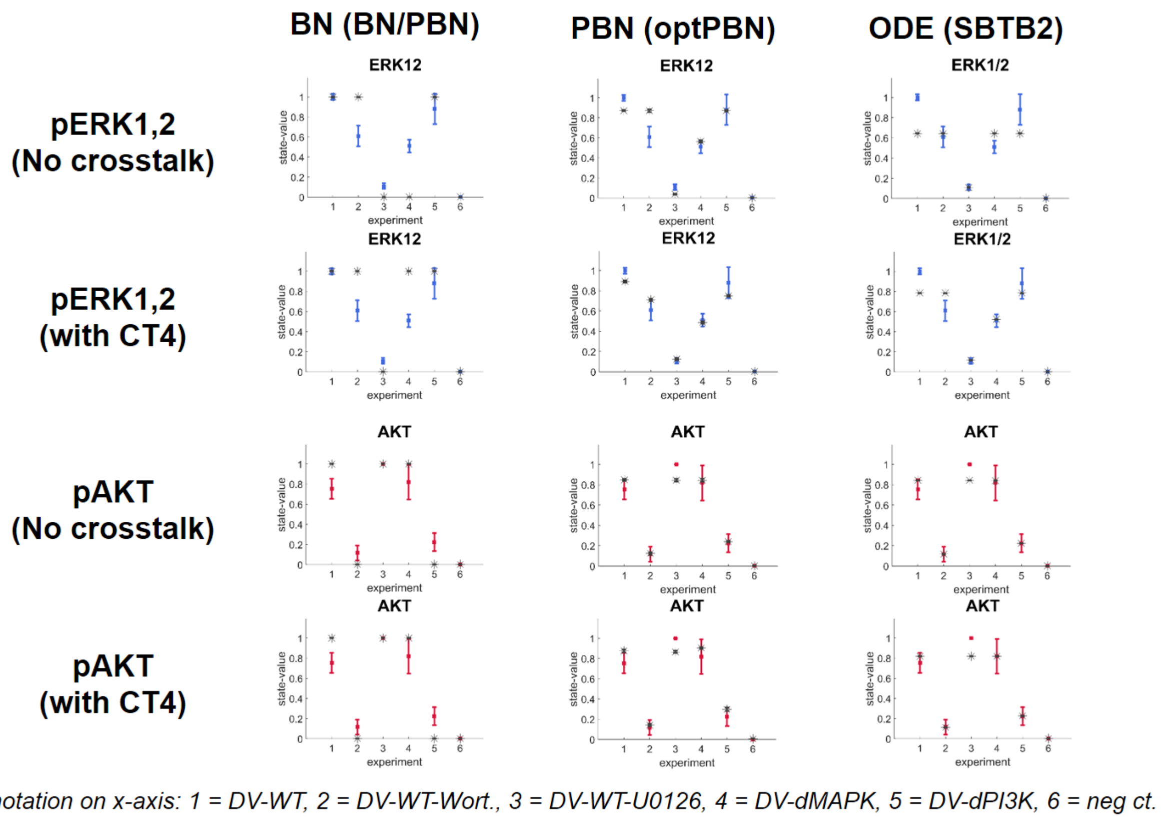

Fig 1. Comparative model fitting among the 3 modelling frameworks

Simulated state values of pERK1,2 and pAKT molecules in two model variants, i.e. the revised model without crosstalk and the final model with essential crosstalk number 4 from PI3K to MEK1,2, generated from the optimised models in 3 different modelling frameworks, i.e. Boolean network (BN), probabilistic Boolean network (PBN) and ordinary differential equation-based model (ODE), are shown.

On the other hand, modelling the same network topology using a detailed mechanistic modelling framework such as ODE-based modelling results in a more complex model structure. One of the contributing factors that increase the complexity is the addition of deactivation/degradation interactions and their corresponding rates for each molecule to balance the activation/synthesis. This assignment is not required in the BN or PBN frameworks. In addition, there are also higher numbers of parameter to be optimised in the ODE-based model.

A comparison of the results of the different modelling approaches (see Table 1) shows that PBN is the most concise modelling approach that still allows to nicely fit the experimental data in our setting with a low number of parameters as also reflected in the respective AIC values. Furthermore, we analysed the distribution of the optimised parameters from 20 optimisation rounds and found that the coefficients of variation of the parameters in the PBN model is less than half of the ones in the ODE-based model (see Tables 2 and 3), pointing to a better identifiability.

Table 2. The distributions of optimised parameters in PBN model.

Rules	Mean	S.D.	C.V.
SHP2 = PDGFR	0.442	0.065	0.147
SHP2 = 0 (dMAPK)	0.558	0.065	0.116
MEK12 = MEK12_induce	0.109	0.057	0.522
MEK12 = 0 (U0126)	0.891	0.057	0.064
PI3K_PDGFR = PDGFR	0.284	0.049	0.172
PI3K_PDGFR = 0 (dPI3K)	0.716	0.049	0.068
PI3K = prePI3K	0.065	0.055	0.844
PI3K = 0 (Wortmannin)	0.935	0.055	0.059
PLCg = PDGFR	0.628	0.048	0.077
PLCg = 0 (dMAPK)	0.372	0.048	0.129
PKC = PKC_induce	0.875	0.055	0.063
PKC = 0 (Wortmannin)	0.125	0.055	0.439
PDGFR = DOX	0.655	0.129	0.197
PDGFR = DOX & ~cCbl	0.046	0.035	0.764
PDGFR = DOX & ~PPX	0.251	0.148	0.591
PDGFR = DOX & ~SHP2	0.048	0.034	0.708
MEK12_induce = Raf1	0.783	0.037	0.047
MEK12_induce = PI3K	0.217	0.037	0.170
PIP3 = PI3K	0.958	0.037	0.039
PIP3 = PI3K & ~bPTEN	0.042	0.037	0.874
PDK = PIP3	0.863	0.156	0.181
PDK = bPDK	0.137	0.156	1.138
AKT = PIP3	0.446	0.256	0.573
AKT = PDK	0.134	0.092	0.685
AKT = MTOR	0.420	0.258	0.614
PKC_induce = IP3_CaIon_DAG	0.017	0.020	1.195
PKC_induce = bPKC	0.983	0.020	0.020
Mean			0.389
Max			1.195

The distributions of optimised parameter values in the PBN model from 20 optimisation runs are shown.

Table 3. Compared distributions of optimised parameters in PBN versus ODE-based models.

Parameters	Mean	S.D.	C.V.
k1	43.482	38.289	0.881
k2	62.307	28.889	0.464
k322	60.359	36.179	0.599
k32	59.426	37.089	0.624
k332	56.388	33.311	0.591
k33	49.547	36.677	0.740
k34	32.001	37.387	1.168
k35	38.040	38.576	1.014
k36	41.857	33.549	0.802
k3	45.452	38.651	0.850
k422	42.145	41.475	0.984
k42	50.923	38.556	0.757
k432	23.979	34.951	1.458
k43	64.362	34.088	0.530
k442	8.018	18.157	2.264
k443	15.123	23.652	1.564
k444	35.469	41.866	1.180
k44	24.888	34.017	1.367
k4	34.011	37.363	1.099
k52	40.836	45.777	1.121
k532	51.611	36.170	0.701
k53	55.524	43.146	0.777
k5	40.195	27.679	0.689
kU0126	29.267	35.028	1.197
kU01262	18.576	34.757	1.871
kWort1	31.265	38.271	1.224
kWort2	44.524	41.426	0.930
kcross4	57.410	40.586	0.707
kdMAPK1	27.236	38.040	1.397
kdMAPK2	0.292	0.015	0.053
kdPI3K	45.648	42.130	0.923
koff1	47.415	40.630	0.857
koff2	21.251	36.930	1.738
koff3	25.331	34.526	1.363
koffppx	52.721	37.922	0.719
kon	48.624	28.877	0.594
kp1	43.146	36.352	0.843
kp2	7.466	5.160	0.691
kp322	57.356	38.308	0.668
kp32	36.276	37.604	1.037
kp33	34.415	35.994	1.046
kp34	36.856	38.707	1.050
kp35	10.310	21.131	2.050
kp36	26.593	23.111	0.869
kp3	41.709	35.960	0.862
kp42	15.970	19.069	1.194
kp43	39.348	40.047	1.018
kp442	43.193	35.014	0.811
kp44	34.093	25.767	0.756
kp4	37.254	44.578	1.197
kp52	45.600	39.275	0.861
kp53	1.980	2.271	1.147
kp5	7.986	5.398	0.676
Mean			0.992
Max			2.264

The distributions of optimised parameter values in the ODE model from 20 optimisation runs are shown.

We also compared the quality of the model predictions of the combinatorial perturbation experiments. The respective predictions for dMAPK with Wortmannin and dPI3K with Wortmannin are shown in Fig 2.

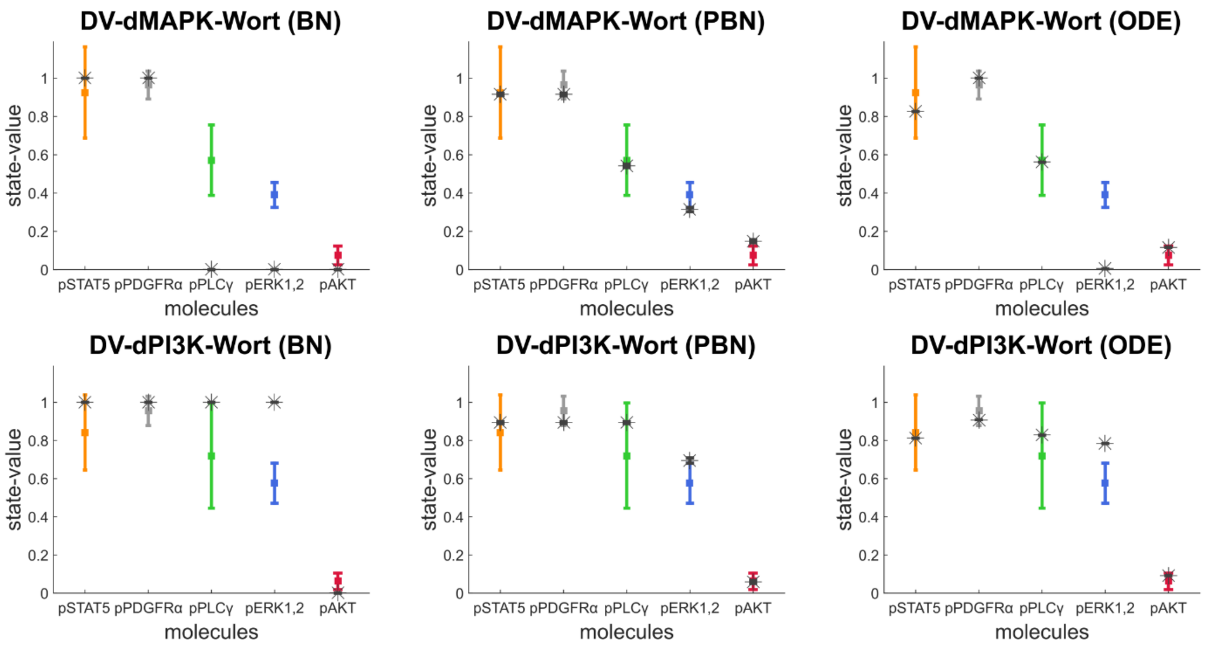

Fig 2. Comparison of model predictions from 3 different modelling approaches.

Model predictions on the signalling profiles of two combined perturbation experiments, i.e. dMAPK with Wortmannin (upper row) and dPI3K with Wortmannin (lower row), generated from 3 different modelling approaches, i.e. Boolean networks (BN), probabilistic Booelan network (PBN), and ODE-based model, are shown. Mean and standard deviation of ten simulated values from the 3 models (black stars [means] and error bars [SD] on top) were compared against the experimental data (multi-coloured squares [mean] and error bars [SD] on bottom). Five molecules as labelled on the x-axis are in the following order: pSTAT5, pPDGFRα, pPLCγ, pERK1,2 and pAKT.

Similar to the quality of model fitting, the quality of model predictions from the Boolean network approach is poor due to inherent qualitative nature of this framework. Even if a discretisation step is applied prior to the comparison, the discretised experimental data of pPLCγ in the DV-dMAPK-Wortmannin condition (1) still does not match the predicted Boolean logic value (0). On the other side, we found that the quality of model predictions from the ODE-based model is generally high, only slightly below the quality of the PBN model predictions.
